# Test-time adaptation for surgical instrument segmentation under visual degradations: selective updating and temporal constraints

**DOI:** 10.3389/frobt.2026.1991989

**Published:** 2026-10-23

**Authors:** Shiqi Jiang, Hai Peng, Shuoxin Gu, Dingqi Zhang, Yupei Zhu, Erteng Zhong, Fulin Wu

**Affiliations:** 1 School of Automation, Chengdu University of Information Technology, Chengdu, China; 2 International Joint Research Center for Robotics and Intelligent Systems, Chengdu, China

**Keywords:** robust visual perception, surgical instrument segmentation, surgical robotics, temporal constraints, test-time adaptation, uncertainty estimation

## Abstract

**Introduction:**

Visual perception for robot-assisted minimally invasive surgery is sensitive to low illumination, smoke, blood interference, and imaging blur, which can degrade segmentation performance during continuous video.

**Methods:**

We propose Lightweight Spatiotemporal Test-Time Adaptation (LiteST-TTA), combining a Top-k predictive-entropy uncertainty gate, motion-compensated historical predictions, hybrid temporal supervision, and updates restricted to Batch Normalization affine parameters. We evaluate five methods on EndoVis 2017 under 12 controlled degradation conditions (four types × three severity levels).

**Results:**

TENT yields a macro-average DSC of 0.8881 and LiteST-TTA yields 0.8879. A significant degradation-type-by-severity interaction is observed for DSC (Greenhouse–Geisser corrected p = 0.031) and DSC improvement over Source (p < 0.001). Under an identical 24.2% update schedule, LiteST-TTA and Selective Entropy achieve nearly identical DSC (0.886967 vs. 0.886966). Statistics-only BN adaptation yields a DSC of 0.8864. In sequence-disjoint evaluation, LiteST-TTA improves severe low-light performance (0.8083 vs. 0.3251 for Source), whereas Source is superior in 10 of 12 held-out conditions.

**Discussion:**

Adaptation benefits depend strongly on degradation type, severity, normalization adaptation, update dynamics, and computational cost. These factors should be considered jointly when applying online adaptation to surgical video.

## 1 Introduction

Robot-assisted minimally invasive surgery is evolving from a mechanical execution platform toward an intelligent medical robotic system that integrates perception, understanding, planning, and interaction. Reliable endoscopic vision is a prerequisite for instrument tracking, field-of-view navigation, tissue-interaction analysis, and potentially semi-autonomous or autonomous operation. Unlike offline medical image analysis, robotic surgical vision must operate in continuously changing video streams. During surgery, camera and instrument motion coexist with pronounced non-rigid tissue deformation, while smoke, blood occlusion, specular reflection, illumination variation, and defocus blur can further alter the visual appearance over time. Consequently, even a source segmentation model that performs well on a standard validation set may degrade during a complete surgical procedure as visual conditions and data distributions change, making stable and reliable perception difficult to maintain.

Semantic segmentation methods represented by U-Net, DeepLab, and lightweight mobile architectures have substantially improved surgical instrument segmentation. However, these approaches generally assume that the training and test distributions are reasonably aligned. When intraoperative images are affected by previously unseen visual degradation, changes in imaging conditions, or device-specific domain shifts, a fixed source model may no longer match the incoming distribution, resulting in lower predictive confidence, inaccurate instrument boundaries, or even missed detections. In robotic surgery, such perception errors are not confined to a single segmentation frame; they can also propagate to downstream instrument localization, motion estimation, interaction-region recognition, and control decisions. Maintaining stable and reliable perception at test time is therefore an important problem for intelligent surgical robotics.

Test-time adaptation (TTA) provides a practical strategy for addressing test-distribution shifts by dynamically adjusting a model during inference using unlabeled test data. Representative methods such as TENT minimize predictive entropy and update normalization-related parameters online so that the model can respond to the statistics of the current test distribution. Subsequent research has extended this idea toward continual TTA, reliable sample selection, and mitigation of model drift. Nevertheless, test-time adaptation does not provide a consistent improvement in every setting. When the test data remain close to the source distribution, unnecessary parameter updates may disturb useful representations already learned by the model. When pseudo-supervision is unreliable or prediction errors accumulate over time, continual adaptation may further induce model drift and compromise long-term inference stability.

Adjacent frames in surgical videos are strongly correlated in time and can therefore provide supervision beyond a single-frame prediction. Although instrument motion, camera displacement, and tissue deformation introduce spatial offsets between neighboring frames, optical-flow-based motion compensation can align a previous prediction with the current frame and provide a temporal reference without labels. Historical predictions, however, may themselves contain errors, and using them directly can propagate incorrect information across frames. Temporal supervision must therefore account for both the reliability of the current prediction and the consistency of the aligned historical prediction in order to reduce the influence of unreliable temporal information during online adaptation.

Based on these considerations, this study extends the objective from maximizing a single average performance measure to examining the applicability, reliability, and computational efficiency of test-time adaptation in complex intraoperative environments. We evaluate how adaptation behaves across different forms and severities of visual degradation, how uncertainty and temporal information influence adaptation stability, and how segmentation accuracy, temporal consistency, update frequency, and inference efficiency interact. We therefore propose Lightweight Spatiotemporal Test-Time Adaptation (LiteST-TTA) for robotic surgical video segmentation, which combines parameter-efficient updating with temporal information to enable selective online adaptation from unlabeled video streams. Using a unified source model and test protocol, we systematically compare LiteST-TTA with Source-only, BN Adapt, TENT, and Selective Entropy across 12 controlled visual degradation settings. The main contributions are summarized as follows:

1. We propose LiteST-TTA for robotic surgical video segmentation. It integrates a Top-k uncertainty gate, a motion-compensated hybrid temporal pseudo-label, and BN-affine parameter updating to perform selective online adaptation by modifying only a small subset of normalization parameters while using adjacent-frame information to strengthen temporal supervision at test time.
2. We establish a controlled intraoperative visual-degradation protocol covering blood interference, blur, low illumination, and smoke at three degradation-specific severity levels. The evaluation forms a repeated factorial structure over degradation type, severity, and adaptation method, and the generation parameters and ranges of all 12 conditions are explicitly reported for reproducibility.
3. We statistically characterize degradation-dependent adaptation behavior rather than relying only on cell-wise visual comparisons. Sequence-level repeated-measures analysis confirms a type-by-severity interaction in both absolute DSC and the DSC gain relative to Source, supporting the conclusion that adaptation effectiveness is jointly conditioned by degradation form and severity.
4. We add mechanism-oriented controls and robustness checks for temporal updating, BN adaptation, and source-checkpoint selection. A matched-update experiment shows that the original LiteST-TTA advantage over Selective Entropy is not preserved when both methods update the same frames, while a BN ablation shows that test-time normalization statistics account for most of the aggregate recovery. A sequence-disjoint sensitivity analysis further separates checkpoint selection from final degradation evaluation and shows that the dominant low-light adaptation pattern persists on completely held-out video sequences. These analyses clarify the attribution and evaluation limits of the proposed pipeline and motivate adaptation-effectiveness-aware gating, lower-cost temporal modeling, and safer fallback strategies.

## 2 Related work

This section reviews research related to robotic surgical vision under complex intraoperative conditions, with an emphasis on surgical instrument segmentation, medical image domain adaptation and test-time adaptation, and temporal modeling for surgical video. Deep learning has markedly advanced pixel-level instrument segmentation, with the field progressing from convolutional neural networks toward vision Transformers and foundation segmentation models. However, most methods optimize segmentation accuracy under a fixed training distribution, and performance can still decline when intraoperative images are affected by illumination changes, smoke occlusion, blood interference, or imaging blur. Domain adaptation and test-time adaptation provide promising ways to mitigate performance degradation under unseen test distributions, yet existing work has focused primarily on shifts across devices, imaging protocols, or medical centers and has made relatively limited use of dynamic visual changes and inter-frame temporal information in continuous surgical video. Conversely, temporal surgical segmentation studies have shown that motion cues and temporal context can improve continuity and stability, but how such information can guide online parameter adaptation without labels remains underexplored.

### 2.1 Robotic surgical vision and instrument segmentation

Detection, tracking, and segmentation of surgical instruments are fundamental components of visual perception in robot-assisted surgery, providing information for instrument localization, motion analysis, surgical scene understanding, and subsequent human-robot collaboration. Bouget et al. (2017) systematically reviewed vision-based markerless surgical tool detection and tracking and highlighted the importance of real-time tool localization and pose information in computer-assisted surgery. Wang et al. (2022) further reviewed visual detection and tracking methods for minimally invasive surgical instruments, emphasizing detection accuracy, processing speed, and robustness to complex scenes. Rueckert et al. (2024) reviewed minimally invasive surgical instrument segmentation in both images and videos and identified temporal information as an increasingly important direction for further development.

With the development of deep convolutional networks, surgical instrument segmentation has progressed from local texture extraction toward joint modeling of multiscale semantics and attention. Ni et al. (2022) proposed SurgiNet, which uses pyramid attention aggregation and class-wise self-distillation to enhance instrument representation under complex illumination. Kurmann et al. (2021) introduced a mask-then-classify strategy for multi-instance instrument segmentation, separating region extraction from instance classification. Yang et al. (2023) proposed MAF-Net, which integrates multiscale attention to strengthen local context and instrument features at different scales. Liu et al. (2024) proposed SINet, combining object detection and semantic segmentation to balance instrument-perception accuracy and real-time efficiency.

More recently, pretrained visual models and foundation segmentation models have been introduced into surgical instrument segmentation to improve feature representation with limited annotations. Wei et al. (2024) used a convolutional network as a lightweight adapter to integrate pretrained Vision Transformer features and improve robustness across surgical scenes. Sheng et al. (2024) proposed Surgical-DeSAM, which automatically generates instrument bounding-box prompts to reduce the manual interaction required by SAM in robotic surgery. Peng et al. (2024) combined active learning with synthetic images to reduce pixel-level annotation requirements and demonstrated the usefulness of synthetic samples for training instrument segmentation models under limited annotation budgets.

Overall, surgical instrument segmentation has expanded from convolutional feature extraction to multiscale attention, Vision Transformers, and foundation segmentation models, with substantial gains in accuracy and runtime on standard public datasets. These methods, however, primarily improve performance through architecture design and supervised training, implicitly assuming that the training distribution adequately covers deployment conditions. Real intraoperative video is highly dynamic, and low illumination, smoke, blood interference, and imaging blur may evolve throughout a procedure, making it difficult for a fixed-parameter model to maintain stable segmentation. Improving source-model representation during training alone is therefore unlikely to fully satisfy the need for continuously reliable perception in complex surgical environments.

### 2.2 Medical image domain adaptation and test-time adaptation

Medical image models are sensitive to differences in imaging devices, acquisition protocols, and institutions, making domain adaptation an important strategy for improving cross-domain generalization. Endoscopic images are similarly affected by changes in surgical devices, illumination, and imaging environments, which can alter the appearance of both instruments and tissue. Sahu et al. (2021) proposed a teacher-student simulation-to-real adaptation method that uses unlabeled real endoscopic images to reduce the domain gap between synthetic data and real surgical scenes. Liu et al. (2022) introduced graph-based adaptive instrument segmentation using domain-common knowledge to improve segmentation in unseen domains. Kumari and Singh (2024) reviewed unsupervised domain adaptation in medical imaging and identified source-target distribution discrepancy as a continuing challenge for practical generalization.

Traditional domain adaptation generally assumes that a target-domain dataset can be collected before deployment and used for additional training or distribution alignment. This assumption becomes restrictive when source images cannot be revisited or the target distribution changes continuously. Karani et al. (2021) proposed test-time adaptable neural networks for medical image segmentation by adapting an image-normalization subnetwork at inference. Bateson et al. (2022) developed source-free domain adaptation for image segmentation using unlabeled target data without revisiting source images. Wu et al. (2023) proposed uncertainty-aware pseudo-label guidance for source-free medical image segmentation to reduce the effect of unreliable supervision, while Stan and Rostami (2024) investigated source-free unsupervised model adaptation that leverages knowledge retained in pretrained models to improve out-of-distribution medical image segmentation.

As test data evolve from a single target domain into a continuous stream, test-time adaptation must additionally address model drift, error accumulation, and catastrophic forgetting. Zhu et al. (2025) proposed continual test-time domain adaptation that combines uncertainty and shape information with stochastic weight resetting to alleviate forgetting under evolving target domains. Zhang et al. (2025) introduced PASS, which jointly learns style and semantic-shape prompts to adapt to appearance and structural changes at test time. Wu et al. (2026) proposed SicTTA, which improves normalization stability in single-image continual test-time adaptation through source-friendly sample selection and feature fusion. (Kim et al., 2026) combined feature augmentation with weight averaging to reduce error accumulation during long-term continual adaptation.

Medical image adaptation has thus progressed from offline target-domain adjustment to source-free and continual test-time adaptation, with increasing attention to uncertainty estimation, pseudo-label reliability, and long-term update stability. These developments provide an important foundation for online adjustment under unknown test distributions, but most existing methods target cross-domain differences among devices, scanning protocols, or medical centers and typically treat medical images as independent samples or generic sequential inputs. Robotic surgical vision has a more explicit video-stream structure: visual degradation may evolve over time, while adjacent frames retain information about instrument motion, tissue deformation, and scene structure. Test-time adaptation strategies developed primarily for static medical images therefore leave room for better use of temporal information in continuous surgical video.

### 2.3 Temporal modeling and online adaptation in surgical video

Adjacent frames in surgical video are highly correlated, and instrument positions and tissue structures generally change continuously over short time intervals. Temporal information can therefore provide constraints beyond single-frame perception and reduce fluctuations caused by local occlusion, imaging noise, or transient degradation. At the same time, camera motion, rapid instrument movement, and non-rigid tissue deformation create substantial spatial displacement between frames, making reliable inter-frame correspondence a central problem in temporal surgical-video modeling.

Sestini et al. (2023) proposed FUN-SIS, which uses instrument motion derived from optical flow to generate pseudo-labels for fully unsupervised surgical instrument segmentation. Their study demonstrates that motion information intrinsic to surgical video can be converted into an additional supervisory signal, offering a way to reduce dependence on pixel-level manual annotation.

Beyond motion cues, cross-frame context and stereo information have been used to improve video segmentation stability. Wang et al. (2025) proposed LACOSTE, which jointly exploits stereo and temporal context to increase robustness to instrument motion and viewpoint changes. The method illustrates how cross-frame feature aggregation can reduce failures of single-frame segmentation under transient appearance variation.

As foundation segmentation models have expanded to video, maintaining segmentation continuity through historical target states and cross-frame attention has emerged as another direction. Sivakumar et al. (2025) proposed SASVi, which updates SAM2 target states through automatic re-prompting to maintain segmentation throughout complete surgical videos. Xue et al. (2026) introduced TD-SAM, which combines dynamic cross-frame attention with distance-guided instance refinement to strengthen temporal modeling in complex surgical video.

Existing studies show that optical-flow motion cues, cross-frame feature aggregation, and video foundation models can all exploit temporal correlations in surgical video to improve segmentation continuity and stability. These approaches, however, primarily introduce temporal structures during training or maintain target states during inference; their objective differs from online parameter adaptation using unlabeled test data. For a source model that has already been trained and deployed in a surgical robotic system, how continuous video information should guide test-time parameter updates remains insufficiently studied.

Historical predictions and motion estimates are also not uniformly reliable. Segmentation errors from a previous frame may propagate after motion compensation, while optical-flow estimation adds computational cost and can be affected by rapid movement and local occlusion. Without a reasonable assessment of the current model state and the need for adaptation, continual parameter adjustment may disturb useful source-model representations. Test-time adaptation for surgical video therefore needs not only to exploit temporal information for unlabeled supervision but also to control the range and frequency of parameter updates to reduce instability caused by unreliable history and over-adaptation.

In summary, surgical instrument segmentation has advanced substantially in spatial representation, multiscale modeling, and temporal video modeling, while medical image TTA provides an important basis for online model adjustment under unknown distributions. The connection between these two lines of research, however, remains limited. Medical image TTA usually considers single images or generic continuous streams and makes limited use of inter-frame motion and historical predictions in surgical video; conversely, temporal instrument segmentation mainly improves prediction through cross-frame modeling and seldom considers online parameter adjustment without test labels. Moreover, selective adaptation commonly uses predictive uncertainty to decide whether to update, although uncertainty does not directly reveal whether an update will improve performance. Explicit motion compensation can add temporal information but also increases computational cost. Motivated by these gaps, LiteST-TTA combines uncertainty-gated online updating with motion-compensated historical predictions and a hybrid temporal constraint, and we systematically analyze segmentation quality, temporal stability, update frequency, and inference efficiency under complex visual degradations.

## 3 LiteST-TTA methods

### 3.1 Overall framework

To improve online adaptation of robotic surgical vision models under complex visual degradation, we develop the LiteST-TTA framework. Starting from a pretrained surgical instrument segmentation model, LiteST-TTA performs online adjustment at test time without accessing ground-truth labels or source-domain training images, using only the current unlabeled frame and information from adjacent frames. In contrast to strategies that update parameters on every test sample, LiteST-TTA assesses the current prediction state and performs optimization only when an update is triggered, thereby limiting unnecessary disturbance to source-model representations. The overall framework is shown in Figure 1.

**FIGURE 1:**
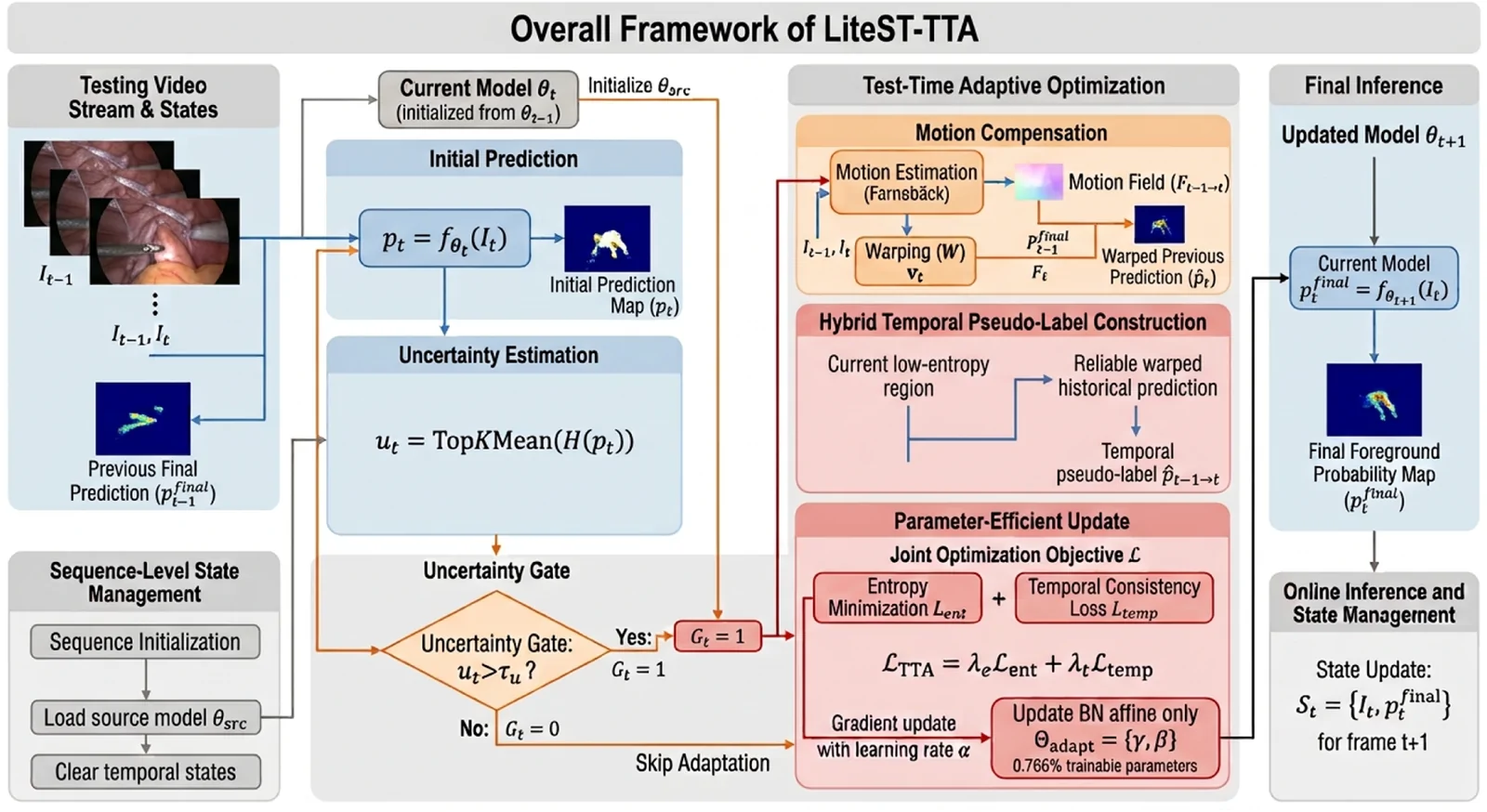
Overall framework of LiteST-TTA.

Let the labeled source-domain training set be denoted as:

$$D_{s}={\left\{\left(x_{i}^{s},y_{i}^{s}\right)\right\}}_{i=1}^{N_{s}}$$ (1)

where $x_{i}^{s}$ denotes a source-domain endoscopic image and $y_{i}^{s}$ is the corresponding pixel-level instrument segmentation label. Supervised training yields the source segmentation model $f_{\theta_{0}}$ , where $\theta_{0}$ denotes the trained model parameters. After deployment, the model receives an unlabeled surgical video sequence in temporal order:

$$X={\left\{x_{t}\right\}}_{t=1}^{T}$$ (2)

Ground-truth segmentation labels are unavailable during testing. Because intraoperative images may be affected by illumination variation, smoke, blood interference, and imaging blur, the test distribution can drift over time relative to the source training distribution, causing a fixed model to exhibit degraded segmentation and unstable predictions across consecutive frames.

The source segmentation network consists of MobileNetV3 and LR-ASPP. For the input $x_{t}$ at frame t , the current model first produces an instrument foreground probability map:

$$p_{t}=f_{\theta_{t}}\left(x_{t}\right)$$ (3)

where $\theta_{t}$ denotes the model state used to process the current frame, and $p_{t}\in {\left[0,1\right]}^{H\times W}$ . Pixel-wise predictive entropy is then used to estimate the uncertainty of the current frame, and the resulting frame-level uncertainty serves as the trigger for online adaptation. When the uncertainty is below a predefined threshold, the current model parameters are retained and the prediction is returned directly. When the threshold is exceeded, the test-time adaptation procedure is activated. Equations 1–3 define the source-domain training set, the test sequence, and the current-frame prediction, respectively.

For a frame that triggers adaptation, LiteST-TTA exploits the temporal continuity of surgical video to construct unlabeled supervision. Dense optical flow is first estimated between the current and previous frames, and the historical prediction is warped into the current coordinate system. Reliable regions are then selected jointly from the confidence of the current prediction and that of the motion-compensated historical prediction to construct hybrid temporal supervision. The online objective combines current-frame entropy with cross-frame temporal consistency, while gradient-based updating is restricted to the affine parameters of Batch Normalization (BN) layers.

After one online update, the adapted model performs a second forward pass on the current frame, and the final probability prediction is stored as the historical state for the next time step. For frames that do not trigger an update, the current prediction is likewise stored in the temporal state so that historical information remains continuous. At the beginning of each independent test sequence, the model parameters are reinitialized to the same source-model state $\theta_{0}$ , limiting the accumulation of adaptation errors across sequences.

The overall online processing pipeline of LiteST-TTA therefore consists of current-frame prediction, uncertainty estimation, selective adaptation, motion compensation, hybrid temporal supervision, BN-affine parameter updating, and temporal-state maintenance. The following subsections describe the key components in detail.

### 3.2 Selective updating based on predictive uncertainty

In continuous test video, the degree of visual degradation varies from frame to frame. Some test frames remain similar to the source-domain data, allowing the source model to maintain stable segmentation. Continuously applying gradient updates to every frame would not only increase computation but could also disturb useful representations already learned by the model. We therefore use the current prediction state to determine whether online adaptation is needed and trigger parameter updates only for frames with a greater apparent need for adaptation.

For binary instrument segmentation, let the predicted foreground probability at pixel i of frame t be $p_{t}\left(i\right)$ . We use normalized binary predictive entropy to quantify pixel-wise uncertainty:

$$\begin{array}{cccc} & H_{t}\left(i\right)=\frac{-\left[p_{t}\left(i\right)\log p_{t}\left(i\right)+\left[1-p_{t}\left(i\right)\right]\log\left[1-p_{t}\left(i\right)\right]\right]}{\log2} & & \end{array}$$ (4)

where $H_{t}\left(i\right)\in \left[0,1\right]$ . When the predicted probability $p_{t}\left(i\right)$ approaches 0 or 1, the corresponding predictive entropy is low. When $p_{t}\left(i\right)$ approaches the binary decision boundary of 0.5, the entropy increases, indicating greater uncertainty at that pixel.

Surgical instruments usually occupy only part of an image, while a large proportion of background pixels are predicted with high confidence. Higher uncertainty is more likely to occur around instrument boundaries, local occlusions, and regions affected by pronounced visual degradation. A simple average over all pixels can therefore dilute these localized high-uncertainty responses with a large number of low-entropy background pixels, making a frame-level score less sensitive to local prediction anomalies. We consequently adopt a Top- k aggregation strategy for frame-level uncertainty.

Let $\Omega_{t}^{k}$ denote the set containing the k pixels with the highest entropy values in the current image. The frame-level uncertainty is defined as:

$$\begin{array}{cccc} & U_{t}=\frac{1}{|\Omega_{t}^{k}|}\sum_{i\in \Omega_{t}^{k}}H_{t}\left(i\right) & & \end{array}$$ (5)

By aggregating the most uncertain regions of the prediction, $U_{t}$ emphasizes local changes associated with visual degradation. Based on this measure, we define a frame-level update gate:

$$\begin{array}{cccc} & G_{t}=I\left(U_{t}>\tau_{u}\right) & & \end{array}$$ (6)

where $\tau_{u}$ is the uncertainty threshold and $I\left(\cdot \right)$ denotes the indicator function. When $G_{t}=0$ , the current frame does not trigger a test-time parameter update; the existing model state is retained and the current prediction is returned directly. When $G_{t}=1$ , online adaptation is triggered and adjacent-frame information is used to construct temporal constraints and the optimization objective. LiteST-TTA therefore updates parameters only on test frames with sufficiently high predictive uncertainty, reducing unnecessary online updates in continuous video. Equations 4–6 define the pixel-wise predictive entropy, frame-level uncertainty, and uncertainty-gated update decision, respectively.

Frame-level uncertainty in this study is used primarily to determine whether the current prediction warrants further adaptation; it is not treated as a direct measure of the performance change caused by the update itself. The relationship among predictive uncertainty, update frequency, and segmentation performance under different visual degradations is therefore examined explicitly in the experimental analysis.

### 3.3 Motion-compensated temporal information

When the current frame triggers online adaptation, constructing the optimization objective only from the current prediction would fail to exploit the temporal correlation between adjacent surgical-video frames. Instrument shape and local tissue structure generally evolve continuously over short time intervals, so the previous prediction can provide a temporal prior for unlabeled adaptation of the current frame. However, camera motion, instrument displacement, and non-rigid tissue deformation cause the same object to shift spatially across consecutive frames. Directly enforcing consistency between unaligned predictions could therefore treat normal motion as a prediction error. We first compensate for inter-frame motion so that the historical prediction is spatially aligned with the current frame.

Given adjacent video frames $I_{t-1}$ and $I_{t}$ , Farneback dense optical flow is used to estimate the two-dimensional motion field from the previous frame to the current frame:

$$\begin{array}{cccc} & F_{t-1\to t}=\Phi \left(I_{t-1},I_{t}\right) & & \end{array}$$ (7)

where $\Phi \left(\cdot \right)$ denotes the optical-flow estimation process and $F_{t-1\to t}$ describes pixel displacement between adjacent frames.

Let the final foreground probability map stored after online processing of the previous frame be $p_{t-1}^{\text{final}}$ . Using the estimated motion field, it is warped into the coordinate system of the current frame:

$$\begin{array}{cccc} & q_{t}=W\left(p_{t-1}^{\text{final}},F_{t-1\to t}\right) & & \end{array}$$ (8)

where $W\left(\cdot \right)$ denotes the spatial warping operation based on the optical-flow field, and $q_{t}$ is the motion-compensated historical prediction aligned with the current frame. In this way, the historical instrument prediction follows inter-frame motion to the corresponding location in the current frame and provides a temporal reference for online adaptation. Equations 7, 8 describe optical-flow estimation and motion-compensated historical-prediction warping, respectively.

Spatial alignment alone does not guarantee that the historical prediction is sufficiently reliable. The previous prediction may already contain segmentation errors, and optical-flow estimation can itself be affected by rapid motion, occlusion, and non-rigid deformation, leaving local misalignment after compensation. We therefore do not use $q_{t}$ as supervision over the entire current frame. Instead, reliable regions are selected using both the current prediction state and the confidence of the aligned historical prediction, and temporal supervision is constructed only within those regions.

### 3.4 Hybrid temporal pseudo-labels and joint adaptation objective

After motion compensation, the historical prediction $q_{t}$ is spatially aligned with the current frame but may still contain errors inherited from the previous prediction or local inaccuracies caused by optical-flow estimation. Using the complete historical probability map as temporal supervision would allow unreliable information to participate in online optimization. LiteST-TTA therefore identifies reliable regions by jointly considering the current prediction and the confidence of the aligned historical prediction, and introduces temporal constraints only where both are considered trustworthy.

For the current prediction, a reliability mask is constructed from pixel-wise predictive entropy:

$$\begin{array}{cccc} & M_{t}^{c}\left(i\right)=I\left(H_{t}\left(i\right)<\tau_{c}\right) & & \end{array}$$ (9)

where $\tau_{c}$ is the low-entropy threshold for the current prediction. Pixels with entropy below this threshold have a clearer foreground/background decision and are retained for subsequent temporal supervision.

For the motion-compensated historical probability $q_{t}\left(i\right)$ , confidence is measured by its distance from the binary decision boundary of 0.5. The corresponding historical reliability region is defined as:

$$\begin{array}{cccc} & M_{t}^{h}\left(i\right)=I\left(|q_{t}\left(i\right)-0.5|>m_{p}\right) & & \end{array}$$ (10)

where $m_{p}$ is the historical confidence margin. Only locations whose motion-compensated historical probabilities lie sufficiently far from 0.5 are retained as reliable historical references.

The current-frame and historical reliability regions are then jointly filtered to obtain the hybrid temporal mask:

$$\begin{array}{cccc} & M_{t}\left(i\right)=M_{t}^{c}\left(i\right)\cdot M_{t}^{h}\left(i\right) & & \end{array}$$ (11)

This mask retains only locations where both the current prediction and the historical prediction are sufficiently confident, reducing the influence of uncertain current predictions and low-confidence historical information on online optimization.

Within the reliable region, the motion-compensated historical probability $q_{t}$ is used as a soft temporal target, and a temporal consistency loss is constructed from the difference between the current prediction $p_{t}$ and the historical prediction:

$$\begin{array}{cccc} & L_{\text{temp}}=\frac{\sum_{i}M_{t}\left(i\right)|p_{t}\left(i\right)-q_{t}\left(i\right)|}{\sum_{i}M_{t}\left(i\right)+\varepsilon } & & \end{array}$$ (12)

where ε is a stabilizing term that prevents division by zero. Compared with binarizing the historical prediction into a hard pseudo-label, probability-based supervision preserves continuous confidence information and imposes a smoother constraint on prediction changes.

In addition to temporal supervision, LiteST-TTA retains an entropy-minimization objective based on the current prediction. The mean predictive entropy of the current frame is defined as:

$$\begin{array}{cccc} & L_{\text{ent}}=\frac{1}{HW}\sum_{i=1}^{HW}H_{t}\left(i\right) & & \end{array}$$ (13)

where H and W denote the height and width of the probability map, respectively. This loss reduces uncertainty in the current prediction and encourages a more decisive foreground-background separation on unlabeled test data.

The entropy-minimization term and the temporal-consistency term are combined into the final online adaptation objective:

$$\begin{array}{cccc} & L_{\text{TTA}}=\lambda_{e}L_{\text{ent}}+\lambda_{t}L_{\text{temp}}, & & \end{array}$$ (14)

where $\lambda_{e}$ and $\lambda_{t}$ control the contributions of current-frame entropy and cross-frame temporal consistency, respectively. The joint objective adapts the model to the current test distribution while constraining the update with motion-compensated historical information, thereby reducing prediction fluctuations that can arise when online optimization relies solely on a single-frame unsupervised objective. Equations 9–14 describe the reliability masks and the temporal, entropy, and joint adaptation objectives.

### 3.5 Parameter-efficient online updating

At test time, the online objective is constructed entirely from the current prediction and temporal information without ground-truth labels. Allowing a large portion of the network to change could amplify the influence of unreliable self-supervision and increase the risk of parameter drift during continuous testing. To limit the effect of a single online adjustment on the source representation, LiteST-TTA restricts gradient-based optimization to the affine parameters of Batch Normalization (BN) layers and freezes the remaining network parameters.

Let the set of BN layers involved in test-time gradient optimization be B . The set of trainable parameters is defined as:

$$\begin{array}{cccc} & \Theta_{\text{adapt}}=\left\{\gamma_{l},\beta_{l}|l\in B\right\} & & \end{array}$$ (15)

where $\gamma_{l}$ and $\beta_{l}$ are the scale and bias parameters of the l -th BN layer, respectively. Relative to the complete model parameter set Θ , we have:

$$\begin{array}{cccc} & |\Theta_{\text{adapt}}|\ll |\Theta | & & \end{array}$$ (16)

Thus, test-time gradient optimization acts only on a small set of normalization affine parameters, while the convolutional layers and the main segmentation network remain fixed. Note that the model operates in training mode during online adaptation, so the BN running mean and running variance are also updated by the incoming test stream; these changes are not produced by gradient descent.

For a test frame satisfying $G_{t}=1$ , one gradient step is applied to the trainable parameters using the joint adaptation objective $L_{\text{TTA}}$ :

$$\begin{array}{cccc} & \Theta_{\text{adapt}}^{t+1}=\Theta_{\text{adapt}}^{t}-\eta \nabla_{\Theta_{\text{adapt}}}L_{\text{TTA}}, & & \end{array}$$ (17)

where η denotes the test-time learning rate. Limiting both the number of updates per frame and the parameter range reduces the influence of continuous online optimization on the main source-model representation. After the update, the adapted model performs another forward pass on the current frame $I_{t}$ to obtain the final prediction:

$$\begin{array}{cccc} & p_{t}^{\text{final}}=f_{\theta_{t+1}}\left(I_{t}\right) & & \end{array}$$ (18)

When the current frame does not meet the update condition, i.e., $G_{t}=0$ , no gradient optimization is performed and the model parameters remain unchanged:

$$\begin{array}{cccc} & \theta_{t+1}=\theta_{t},p_{t}^{\text{final}}=p_{t} & & \end{array}$$ (19)

LiteST-TTA therefore performs local parameter adjustment only on frames that satisfy the uncertainty gate and limits the scope of gradient updates to reduce excessive modification of the source model by unlabeled online optimization. A small trainable parameter subset, however, does not imply the lowest end-to-end inference cost. LiteST-TTA additionally requires motion estimation, historical prediction warping, and a second forward pass after updating; its practical computational efficiency is therefore evaluated using per-frame latency in the experiments. Equations 15–19 define the trainable BN parameters and the corresponding online update rules.

### 3.6 Online inference and sequence-level state management

LiteST-TTA processes continuous surgical video online and must therefore maintain both a changing model state and historical information from adjacent frames. To prevent adaptation states from one video sequence from affecting another, each video sequence is treated as an independent online-processing unit, and the same source-model parameters are reloaded at the beginning of every new sequence:

$$\begin{array}{cccc} & \theta_{1}=\theta_{0} & & \end{array}$$ (20)

where $\theta_{0}$ denotes the trained source-model parameters. At initialization, the image and prediction states retained from the previous sequence are cleared so that every test sequence begins from the same model state.

For the first frame of a sequence, no cross-frame temporal constraint is constructed because no historical image or prediction is available. After processing the first frame, its input image and final prediction are stored as the initial temporal state. When t>1 , the model first applies the current parameters $\theta_{t}$ to the input frame $I_{t}$ to obtain the initial prediction $p_{t}$ , and the frame-level uncertainty $U_{t}$ is calculated from Top- k predictive entropy. If $G_{t}=0$ , test-time gradient updating is skipped and the initial prediction is used as the final output for the current frame. If $G_{t}=1$ , a motion field is estimated between the current and previous frames, the previously stored final prediction is warped to the current frame, temporal supervision is constructed within reliable regions, and one online parameter update is performed.

Regardless of whether an update is triggered, the processed input image and final prediction are stored as the new temporal state:

$$\begin{array}{cccc} & S_{t}=\left\{I_{t},p_{t}^{\text{final}}\right\} & & \end{array}$$ (21)

This state is used at the next time step t+1 for adjacent-frame motion estimation and historical prediction alignment. Historical information therefore propagates continuously within the same video sequence, while model parameters and temporal states do not accumulate across independent sequences.

For a test frame that triggers online adaptation, the main LiteST-TTA processing chain can be summarized as:

$$\begin{array}{cccc} & I_{t}\to p_{t}\to U_{t}\to G_{t}\to q_{t}\to M_{t}\to L_{\text{TTA}}\to \theta_{t+1}\to p_{t}^{\text{final}}\to S_{t} & & \end{array}$$ (22)

When $G_{t}=0$ , motion compensation, temporal supervision construction, and gradient updating are skipped, satisfying $\theta_{t+1}=\theta_{t},p_{t}^{\text{final}}=p_{t}.$

Through this sequence-level state-management strategy, LiteST-TTA preserves a continuous model state and historical prediction information within each video sequence while restoring the same source-model state between independent sequences, thereby forming an online test-time adaptation process for continuous unlabeled surgical video. Equations 20–22 summarize the sequence initialization, temporal-state maintenance, and online processing procedure.

## 4 Experimental setup

### 4.1 Dataset and evaluation protocol

Experiments are conducted on the EndoVis2017 robot-assisted surgical instrument segmentation dataset. We use 1,800 pixel-wise labeled images for source-model training and 901 validation images, originating from 10 video sequences, as the common basis for the original controlled evaluation. The validation images are not used for gradient-based source-model optimization; however, their clean-image labels are used for checkpoint selection as described in Section 4.2. Because the same underlying validation content is subsequently used to generate the original controlled degraded conditions, this main protocol contains model-selection overlap between source-checkpoint selection and evaluation content. We therefore retain the original 901-frame experiment for direct comparison across identical image content and add a separate sequence-disjoint sensitivity analysis in which checkpoint selection and final evaluation use non-overlapping complete video sequences.

To increase the diversity of source training data, all 1,800 original training images are retained and 2,299 additional augmented samples are generated, resulting in 4,099 training instances for the source model. Spatial transformations are applied synchronously to each input image and its pixel-level segmentation label to preserve image-mask correspondence.

At test time, four types of controlled visual degradation—blood interference, imaging blur, low illumination, and smoke—are generated from the same fixed set of 901 validation images. Each degradation is assigned three severity levels (L1, L2, and L3), producing a 4 × 3 set of 12 controlled degradation cells for each of the five evaluated methods. The same 901 frames are used within every cell, yielding a repeated factorial evaluation in which degradation type and within-type severity can be analyzed while holding the underlying image content constant. The labels L1-L3 are ordinal levels defined separately for each degradation and should not be interpreted as a common physical severity scale across Blood, Blur, Low-light, and Smoke. This protocol is designed as a reproducible controlled stress test and is not intended to reproduce the full range of variation encountered in real surgical environments. Figure 2 summarizes the training-data construction and controlled-degradation evaluation protocol.

**FIGURE 2:**
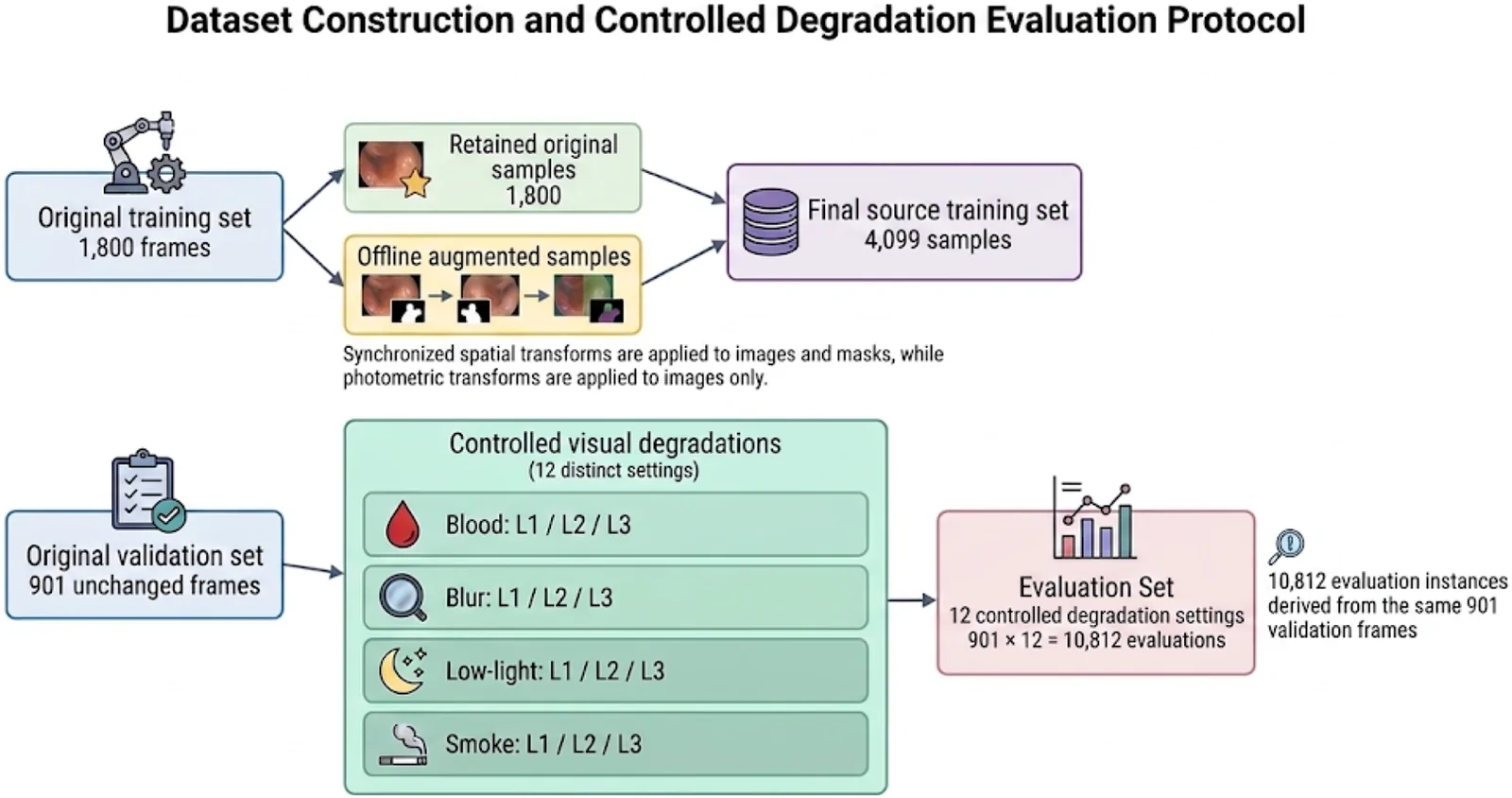
Dataset construction and controlled visual-degradation evaluation protocol.

Figure 3 consolidates the complete evaluation design, including the degradation types, degradation-specific severity settings, adaptation methods, measured responses, and repeated-measures structure.

**FIGURE 3:**
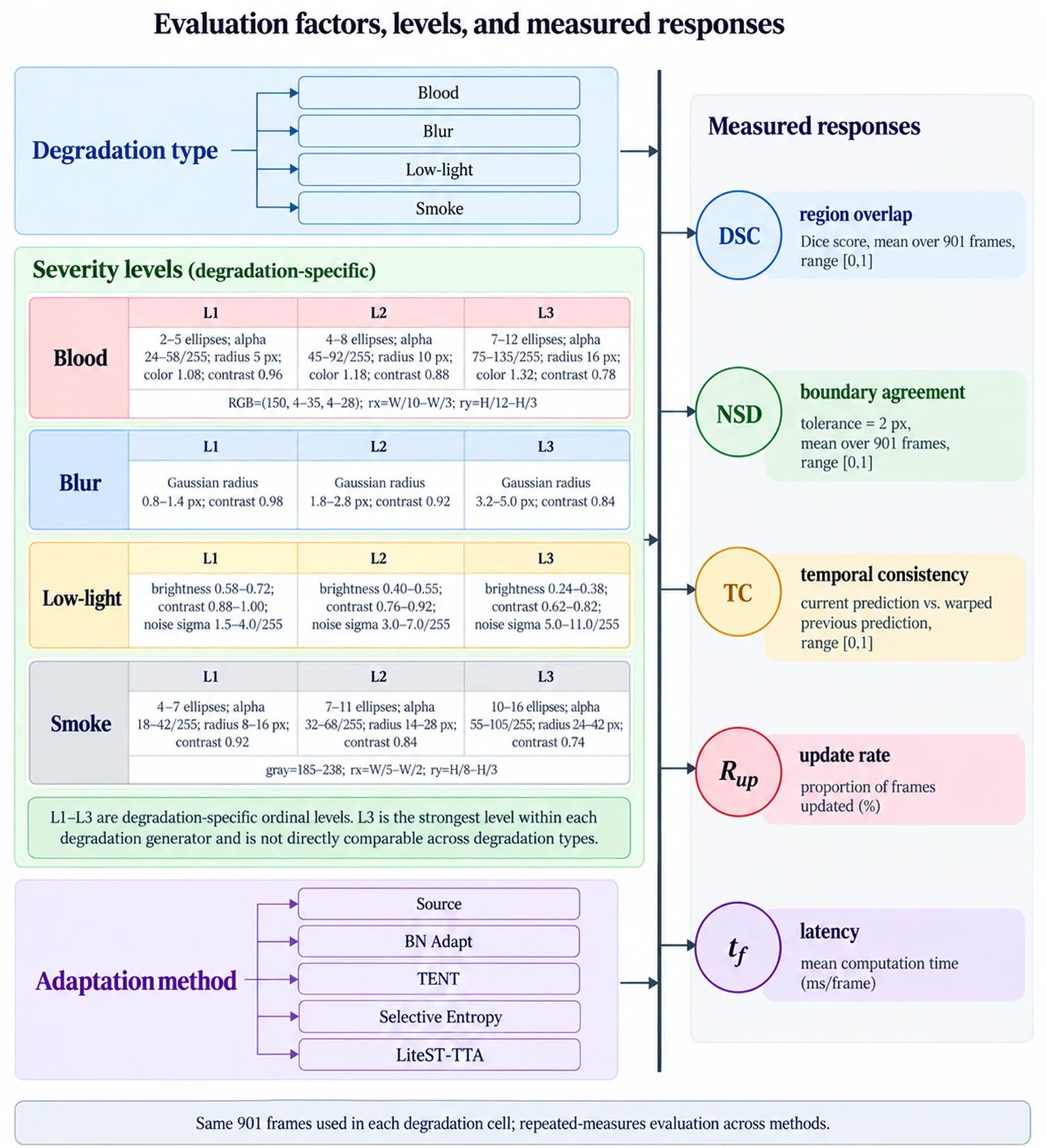
Evaluation factors, degradation-specific severity levels, adaptation methods, and measured responses.

### 4.2 Source-model training and visual-degradation construction

The source model is LRASPP-MobileNetV3-Large. The MobileNetV3-Large encoder is initialized with ImageNet-pretrained weights, whereas the LR-ASPP segmentation head is initialized randomly. Supervised learning is performed once on the source training set, and the resulting best checkpoint is used to initialize Source, BN Adapt, TENT, Selective Entropy, and LiteST-TTA so that all test-time adaptation methods are compared from the same source model.

Training images are resized to 320×320 pixels and the batch size is set to 2. The source model is optimized with AdamW for 40 epochs, using an initial learning rate and weight decay of $1\times 10^{-4}$ . The training objective combines binary cross-entropy and Dice losses. Offline data augmentation is applied only to the source training set; spatial transformations are synchronized between input images and segmentation masks to preserve pixel-level correspondence. Figure 4 shows representative original and augmented images together with their segmentation labels.

**FIGURE 4:**
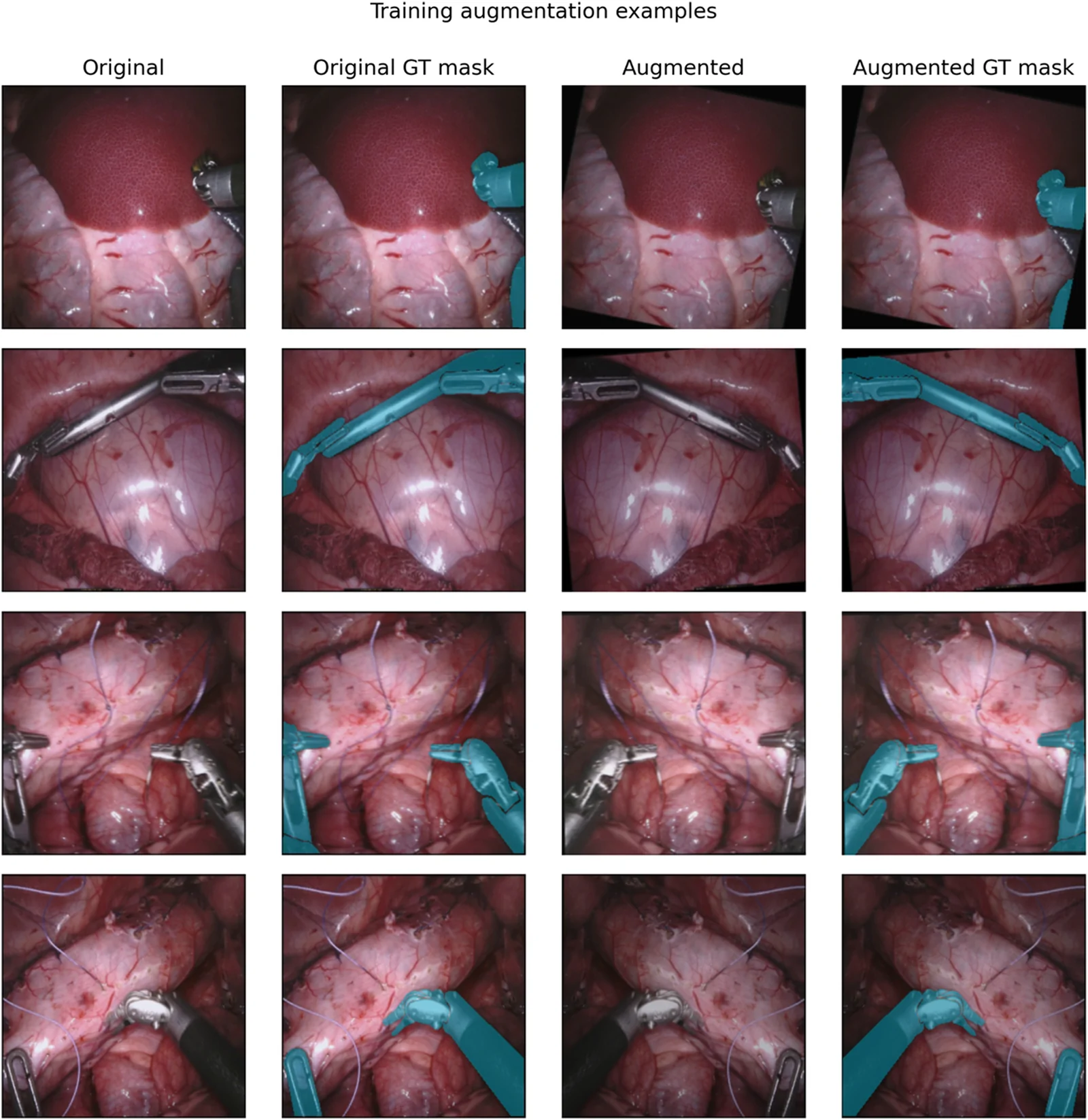
Representative source-domain training augmentations.

For the original 901-frame controlled evaluation, model selection is based on the Dice similarity coefficient (DSC) measured on the fixed clean validation set. The validation DSC reaches its maximum value of 0.9168 at epoch 34, and the corresponding checkpoint is therefore used as the common source model for the original main experiments. Once selected, the source model is not retrained or fine-tuned for any controlled degradation condition. Because checkpoint selection and controlled degradation evaluation share the same underlying validation content, the original Source baseline may benefit from model-selection dependence toward these scene contents even though the validation images are not used for gradient-based training. All adaptation methods are initialized from the same checkpoint, but this common initialization does not by itself remove the potential bias in comparisons between adapted models and the fixed Source baseline. Figure 5 shows the training-loss and validation-DSC trajectories.

**FIGURE 5:**
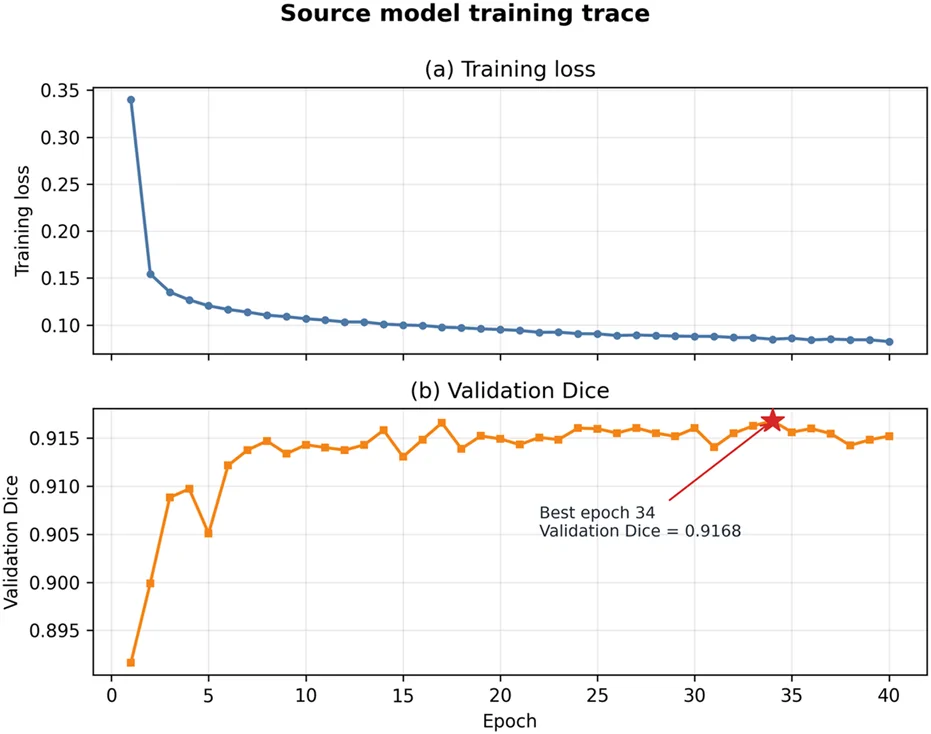
Training trajectory of the source model.

To evaluate the magnitude of this dependence without introducing a different dataset, we additionally perform a sequence-disjoint checkpoint-selection sensitivity analysis. Before checkpoint scoring, the 10 validation video sequences are partitioned at the sequence level using a fixed seed of 42. Sequences 8, 4, 3, 9, and 6 (301 clean frames) are reserved exclusively for checkpoint selection, whereas sequences 7, 10, 5, 1, and 2 (600 clean frames) are reserved exclusively for held-out evaluation. The two groups contain no overlapping sequence. The split is locked before checkpoint scoring and is not changed after inspection of the results.

For this sensitivity analysis, the source model is retrained once using the original model architecture, training data, optimizer, learning rate, weight decay, augmentation settings, and random seed, while all 40 epoch checkpoints are retained. The source checkpoint is selected using only the clean checkpoint-selection sequences. After selection, the chosen checkpoint is frozen before any held-out degradation evaluation is performed. Source, BN Adapt, TENT, Selective Entropy, and LiteST-TTA are then evaluated only on the 12 predefined degradation conditions derived from the five held-out sequences, with the degradation-generation parameters and test-time method settings unchanged.

To evaluate model behavior under complex intraoperative visual changes, we construct four controlled degradations during testing: blood interference, imaging blur, low illumination, and smoke. Blood interference is used to reproduce local color and tissue-appearance changes associated with blood coverage; blur represents loss of image detail caused by defocus or motion; low illumination represents insufficient field brightness; and smoke represents low-contrast occlusion associated with energy-device use.

Each visual degradation is assigned three severity levels, L1, L2, and L3, from mild to severe within that degradation type. Severity is implemented by changing degradation-specific digital image-generation parameters while keeping the underlying frame content and evaluation procedure fixed. The levels are therefore comparable within a degradation type but are not assumed to represent an equivalent physical amount of degradation across different types. The complete generation parameters are reported in Table 1 and Figure 6 provides representative visual examples of the L1-to-L3 progression.

**TABLE 1:** Controlled visual-degradation factors and severity-level generation parameters.

Degradation	Severity	Digital generation parameters
Blood	L1	ellipses = 2–5; alpha = 24–58/255; Gaussian radius = 5 px; color factor = 1.08; contrast = 0.96; RGB=(150,4–35,4–28); rx = W/10-W/3, ry = H/12-H/3
Blood	L2	ellipses = 4–8; alpha = 45–92/255; Gaussian radius = 10 px; color factor = 1.18; contrast = 0.88; RGB=(150,4–35,4–28); rx = W/10-W/3, ry = H/12-H/3
Blood	L3	ellipses = 7–12; alpha = 75–135/255; Gaussian radius = 16 px; color factor = 1.32; contrast = 0.78; RGB=(150,4–35,4–28); rx = W/10-W/3, ry = H/12-H/3
Blur	L1	Gaussian radius = 0.8–1.4 px; contrast = 0.98
Blur	L2	Gaussian radius = 1.8–2.8 px; contrast = 0.92
Blur	L3	Gaussian radius = 3.2–5.0 px; contrast = 0.84
Low-light	L1	brightness = 0.58–0.72; contrast = 0.88–1.00; Gaussian noise sigma = 1.5–4.0/255
Low-light	L2	brightness = 0.40–0.55; contrast = 0.76–0.92; Gaussian noise sigma = 3.0–7.0/255
Low-light	L3	brightness = 0.24–0.38; contrast = 0.62–0.82; Gaussian noise sigma = 5.0–11.0/255
Smoke	L1	ellipses = 4–7; alpha = 18–42/255; Gaussian radius = 8–16 px; contrast = 0.92; gray = 185–238; ellipse rx = W/5-W/2, ry = H/8-H/3
Smoke	L2	ellipses = 7–11; alpha = 32–68/255; Gaussian radius = 14–28 px; contrast = 0.84; gray = 185–238; ellipse rx = W/5-W/2, ry = H/8-H/3
Smoke	L3	ellipses = 10–16; alpha = 55–105/255; Gaussian radius = 24–42 px; contrast = 0.74; gray = 185–238; ellipse rx = W/5-W/2, ry = H/8-H/3

The values in Table 1 are parameters of the digital degradation generator rather than calibrated physical quantities such as lux, optical-density units, or point-spread-function measurements. Accordingly, L3 denotes the strongest level within each degradation generator and is not directly comparable across degradation types.

**FIGURE 6:**
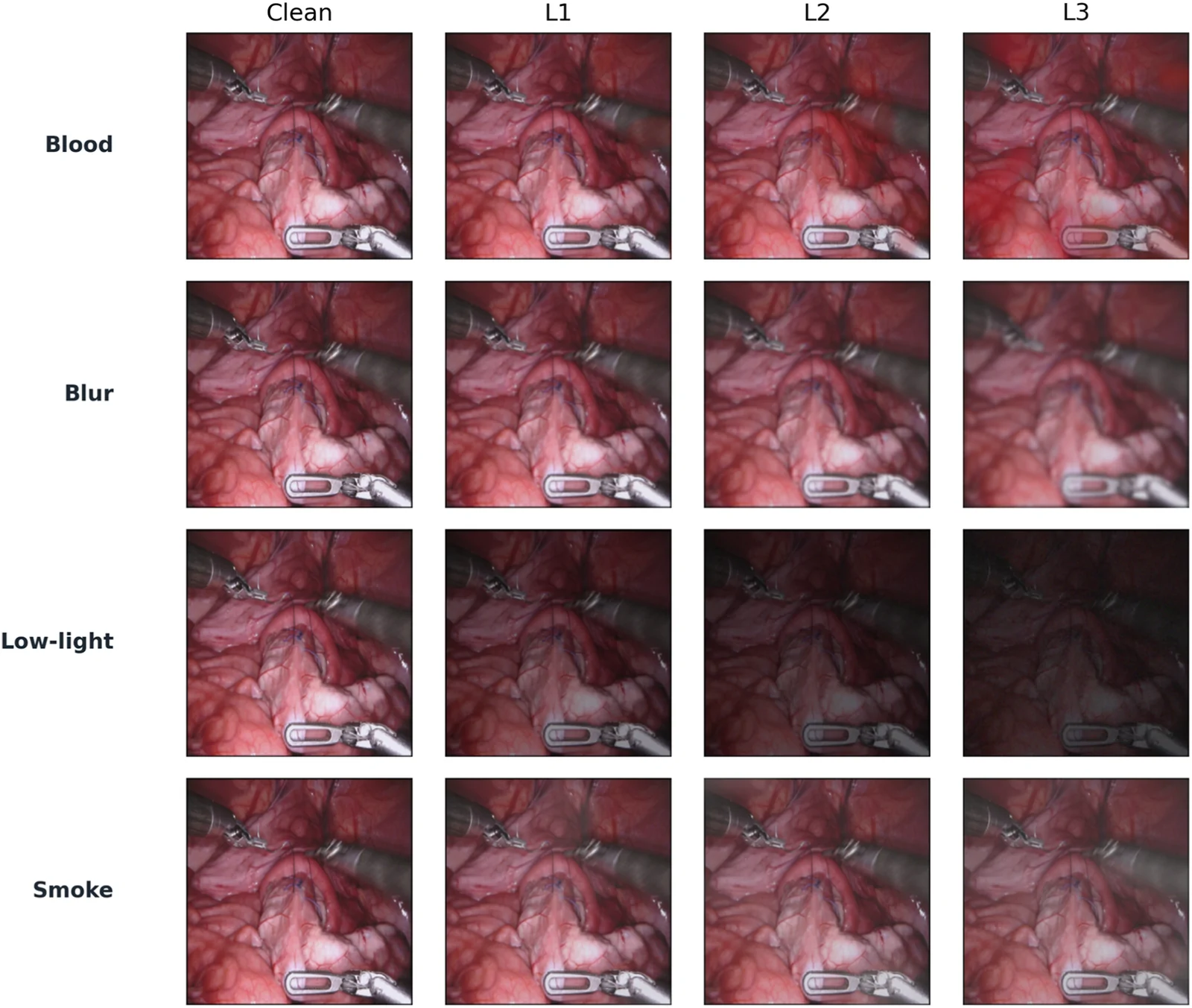
Representative controlled intraoperative visual degradations at three severity levels.

All controlled degradation images were generated once offline before any method evaluation using a fixed global seed of 20,260,902. For corruption index c in {0, 1, 2, three} in the fixed order Smoke, Blood, Low-light, and Blur; severity level l in {1, 2, 3}; and zero-based validation-row index i in {0, 900}, the frame-specific seed was s = 20,260,902 + 1,000,000c + 100,000l + i. Python random. Random(s) generated the per-frame parameters; for Low-light, a derived noise seed was additionally passed to NumPy default_rng to generate the pixelwise noise array. The 10,812 degraded images were saved before evaluation, and Source, BN Adapt, TENT, Selective Entropy, and LiteST-TTA all used the same pre-generated files in the same order. The generation code and a manifest containing the exact per-frame parameter values reconstructed from this deterministic procedure are provided in the accompanying Supplementary Material.

### 4.3 Comparison methods and ablation settings

To compare test-time adaptation strategies under controlled visual degradation, we evaluate five methods—Source, BN Adapt, TENT, Selective Entropy, and LiteST-TTA—using the same source model, preprocessing, and test-frame order. These methods respectively represent fixed inference, normalization-statistics adaptation, continual entropy minimization, selective entropy minimization, and the proposed selective temporal adaptation strategy.

Source directly uses the trained source model for inference and keeps both model parameters and Batch Normalization (BN) statistics fixed throughout testing. It provides the baseline segmentation performance of the unadapted source model under each visual degradation.

BN Adapt places the model in training mode during testing so that the BN running mean and running variance are updated by incoming test data, but no loss-based gradient optimization is performed. This baseline isolates the effect of test-time normalization-statistics adaptation.

TENT uses predictive entropy minimization as the unsupervised test-time objective and performs online parameter updates on continuously arriving frames. It serves as a continual TTA baseline for comparison with selective updating. For a fair comparison of update capacity, gradient optimization is restricted to BN affine parameters.

Selective Entropy augments entropy minimization with a frame-level uncertainty gate and updates parameters only on test frames that satisfy the trigger condition. It does not use historical information from adjacent frames and is therefore the closest structural baseline to LiteST-TTA. However, the gate is state dependent: once the two methods perform different updates, their model states and subsequent entropy trajectories can diverge, so an identical gate threshold does not guarantee identical update frames or update counts. We therefore complement the original comparison with a matched-update control in which both methods update exactly the same frames.

LiteST-TTA extends selective updating by constructing temporal information from motion-compensated historical predictions and combining reliable regions from the current and historical predictions into a hybrid temporal constraint. Entropy minimization and temporal consistency jointly drive online optimization, and gradient updates are likewise restricted to BN affine parameters.

All methods use identical source weights and the same order of test frames. For methods with online adaptation, frames within each video sequence are processed in their original temporal order while the current model state is retained. When a new independent video sequence begins, the common source-model parameters are reloaded and the temporal history is cleared so that adaptation states do not propagate across sequences.

In addition to the main method comparison, component ablations are conducted for the temporal supervision strategy and rollback mechanism in LiteST-TTA. Two further mechanism controls are included. First, a matched-update experiment forces Selective Entropy and LiteST-TTA to follow the same fixed periodic schedule: the first frame of each sequence is not updated and every fourth subsequent frame is updated, thereby matching both update count and update-frame identity. Second, BN adaptation is decomposed into statistics-only adaptation, affine-only optimization with frozen source running statistics, and joint statistics-plus-affine adaptation. These controls are used to separate temporal-objective effects from update-frequency effects and to quantify the contribution of BN statistics to the final behavior.

For the dedicated BN mechanism control, the three modes are implemented explicitly. In the statistics-only configuration, the model is evaluated in training mode under no-gradient inference, so current test-batch statistics are used for normalization and the BN running state is updated by the incoming stream, while no affine parameter is optimized. In the affine-only configuration, the model and BN layers remain in evaluation mode, the source running mean and variance are therefore frozen and used during the forward pass, and gradients are enabled only for the BN affine parameters γ and β. The joint statistics-plus-affine configuration uses test-time BN statistics together with gradient-based optimization of γ and β. This implementation separates normalization-statistics adaptation from affine-parameter optimization.

### 4.4 Evaluation metrics

We evaluate the methods in terms of region-level segmentation accuracy, boundary agreement, temporal stability in video, and online computational efficiency.

Region-level segmentation performance is measured using the Dice similarity coefficient (DSC). Let the predicted instrument region be P and the ground-truth region be G . DSC is defined as:

$$\begin{array}{cccc} & \text{DSC}=\frac{2|P\cap G|}{|P|+|G|} & & \end{array}$$ (23)

DSC takes values in $\left[0,1\right]$ ; a larger value indicates greater overlap between the predicted and ground-truth instrument regions.

To further assess instrument-boundary quality, we use the Normalized Surface Dice (NSD) to measure the spatial agreement between predicted and ground-truth boundaries. Compared with DSC, which primarily measures region overlap, NSD is more sensitive to local boundary deviations and therefore provides a complementary surface-distance-based assessment. A larger NSD indicates closer agreement between the predicted and ground-truth instrument boundaries.

NSD is computed after binarizing the model foreground probability at a threshold of 0.5 and the ground-truth mask at 0.5. Surface points are extracted from the 320 × 320 evaluation masks, and a predicted or ground-truth surface point is counted as matched when its minimum Euclidean distance to the opposite surface is no greater than 2.0 pixels. The reported NSD is the fraction of matched surface points over the total number of predicted and ground-truth surface points; therefore, the NSD tolerance used throughout this study is 2.0 pixels at the resized evaluation resolution.

Because robotic surgical vision operates on continuous video streams, single-frame segmentation metrics alone do not fully characterize temporal prediction stability. We therefore additionally report Temporal Consistency (TC), which measures agreement between the current prediction and the previous prediction after motion compensation. A larger TC indicates stronger agreement across adjacent frames. Because LiteST-TTA optimizes a temporal-consistency loss that uses the same motion-compensated historical prediction mechanism, TC is treated as an optimization-related diagnostic rather than fully independent evidence of temporal improvement.

Specifically, let B_t = 1 [P_t ≥ 0.5] denote the binary prediction for the current frame and let $\overset{∼}{B}$ _(t−1→t) = 1 [W(P_(t−1), F_(t−1→t)) ≥ 0.5] denote the previous foreground-probability map after Farneback optical-flow warping into the current-frame coordinates and thresholding at 0.5. TC is computed as the binary intersection-over-union (IoU) between these two masks: TC_t_ = (|B_t_ ∩ $\overset{∼}{B}$ _t-1_→_t_| + ε)/(|B_t_ ∪ $\overset{∼}{B}$ _t-1_→_t_| + ε), ε = 10^–6^.

The first frame of each video sequence has no historical reference and is recorded as NaN; condition-level TC is averaged only over valid adjacent-frame pairs using NaN exclusion. TC lies in [0,1], with larger values indicating stronger motion-compensated overlap between consecutive binary predictions. Accordingly, the macro-average TC value of 0.388700 for LiteST-TTA represents a mean motion-aligned adjacent-frame IoU of 0.388700 under this definition and should not be interpreted as the percentage of temporally correct frames. No photometric-reliability or confidence mask is applied to the TC evaluation metric itself.

To provide an additional temporal-stability measure that is independent of the optical-flow-based adaptation loss, we also compute the absolute change in ground-truth DSC between consecutive frames within each video sequence. Sequence boundaries are excluded when forming adjacent-frame differences. For each method and degradation condition, the mean and standard deviation of |DSCt−DSCt−1| are reported in Supplementary Table S2; lower values indicate smaller frame-to-frame fluctuations in segmentation accuracy. This label-based stability measure does not use optical flow or historical predictions.

Test-time adaptation also introduces online updates and additional computation. We therefore report both Update Rate and average processing Latency. Update Rate is defined as the fraction of evaluated frames on which online parameter optimization is actually performed:

$$\begin{array}{cccc} & R_{\text{update}}=\frac{N_{\text{update}}}{N_{\text{test}}}\times 100\%, & & \end{array}$$ (24)

where $N_{\text{update}}$ is the number of test frames on which an update is executed and $N_{\text{test}}$ is the total number of evaluated frames.

Average latency is reported in ms/frame and measures the mean processing time required for per-frame inference together with the corresponding test-time adaptation procedure. Joint analysis of DSC, NSD, TC, Update Rate, and Latency characterizes each method from the perspectives of segmentation accuracy, boundary quality, temporal stability, adaptation frequency, and computational efficiency. The DSC and update-rate metrics are defined in Equations 23, 24, respectively.

### 4.5 Implementation details and parameter settings

All methods use the same preprocessing, source-model initialization, and order of test frames. Input images are resized to 320×320 pixels and the test-time batch size is 1. For methods involving online updating, each video sequence is processed in its original temporal order and adapted parameters are retained within the sequence. At the start of a new video sequence, the model is restored to the common source parameters to prevent adaptation states from leaking across independent sequences.

LiteST-TTA uses Adam for test-time parameter optimization with a learning rate of 5 × 10^−5^ and weight decay of 0. At most one gradient step is performed for each triggered frame, with gradient clipping set to 1.0. Gradient optimization is restricted to the BN affine parameters γ and β, corresponding to 24,656 trainable parameters out of 3,218,138 total parameters (0.766%). During online adaptation, the BN layers operate in training mode: current test-batch statistics are used for normalization and the running mean and running variance are updated by the incoming test stream. These BN-state updates are distinct from gradient descent on γ and β.

Frame-level uncertainty is computed from the 20% highest-entropy pixels. At an input size of 320 × 320, this corresponds to 20,480 high-entropy pixels per frame. The uncertainty-gate threshold is τu = 0.08. The weights of the entropy-minimization and temporal-consistency terms are λe = 0.7 and λt = 0.5, respectively. In the hybrid temporal mask, the current-frame low-entropy threshold is τc = 0.35 and the historical-prediction confidence margin is mp = 0.25; thus, a warped historical probability no greater than 0.25 or no smaller than 0.75 is treated as relatively reliable. The photometric-reliability parameter is set to 0.15.

Photometric reliability is an active part of the final temporal-loss implementation rather than an unused configuration parameter. Let Ĩ_(t−1→t) denote the previous denormalized RGB frame warped into the current-frame coordinates using the same Farneback optical flow. For pixel i, the mean absolute RGB residual r_t(i) and photometric reliability R_t(i) are defined as:r_t_(i) = (1/3) Σc |I_t_^c^(i) − Ĩ_t-1_→_t_^c^(i)|, R_t_(i) = exp (−r_t_(i)/σ_p_), σ_p_ = 0.15.

The temporal-consistency loss uses the product of the prediction-confidence term and R_t(i) as its pixel-wise weight; therefore, pixels with larger photometric residuals contribute less to temporal optimization. The parameter σ_p = 0.15 controls the decay scale of this reliability weighting. This photometric reliability is used only in the temporal adaptation loss and is not applied when computing the TC evaluation metric.

Adjacent-frame motion compensation uses Farneback dense optical flow with pyr_scale = 0.5, levels = 3, winsize = 15, iterations = 3, poly_n = 5, and poly_sigma = 1.2. Historical probability maps are warped using bilinear interpolation with replicated borders for samples outside the image. The final main configuration uses the hybrid temporal mask and disables rollback; rollback is evaluated only in the ablation study. The principal LiteST-TTA parameters are summarized in Table 2.

**TABLE 2:** Main test-time adaptation parameters of LiteST-TTA.

Parameter	Setting
Source model	LRASPP-MobileNetV3-large
Input size	320 × 320
TTA batch size	1
TTA optimizer	Adam
TTA learning rate	5 × 10^−5^
TTA weight decay	0
Gradient clipping	1.0
Update steps	1 step per triggered frame
Updated parameters	BN affine parameters
Trainable parameter ratio	0.766%
Top-k uncertainty ratio	20%
Uncertainty threshold	0.08
Current entropy threshold	0.35
Previous confidence margin	0.25
Photometric reliability scale σp	0.15
Entropy weight	0.7
Temporal weight	0.5
Temporal mask	Hybrid
Rollback	Disabled

Latency was measured on an NVIDIA, GeForce RTX, 4060 Laptop GPU, with an Intel Core i7-14650HX CPU, using Python 3.10.20, PyTorch 2.12.1+cu126, and CUDA, 12.6. All methods were evaluated at 320 × 320 resolution with batch size 1 over the complete 10,812-frame controlled evaluation. CUDA, synchronization was performed immediately before and after the timed model/adaptation computation for each frame. The timing excludes image loading, degradation generation, ground-truth mask loading, DSC/NSD/TC, computation, and CSV, writing, while including all method-specific model inference and adaptation operations. For LiteST-TTA, the timed region includes the initial forward pass, dense optical-flow estimation, temporal-supervision and loss construction on triggered frames, gradient-based updating, and the final forward pass. No separate warm-up frames were discarded; the reported values therefore represent observed per-frame computation/adaptation latency under the experimental implementation.

### 4.6 Statistical analysis

The controlled evaluation contains repeated observations of the same underlying video content. To test the central degradation-dependence hypothesis while reducing pseudo-replication from adjacent frames, the primary factorial analysis treats the 10 video sequences as repeated-measure subjects. Two-way repeated-measures ANOVA is performed for LiteST-TTA with degradation type (Blood, Blur, Low-light, and Smoke) and severity (L1-L3) as within-subject factors. The analysis is applied to both absolute DSC and the DSC change relative to Source (ΔDSC). Greenhouse-Geisser-corrected p values are reported together with F statistics and partial eta squared. A frame-level repeated-measures analysis is retained only as a sensitivity analysis.

For condition-wise method comparisons in the original 901-frame evaluation, image-level DSC is summarized as mean ± standard deviation over 901 frames. LiteST-TTA is compared with Source, BN Adapt, TENT, and Selective Entropy using paired Wilcoxon signed-rank tests and paired t tests, with 95% confidence intervals and Cohen’s dz reported for the paired differences. Wilcoxon p values are adjusted across the 12 degradation cells for each comparison method using the Holm procedure. Because adjacent frames are correlated, sequence-level Wilcoxon tests are additionally used as exploratory sensitivity analyses with the 10 video sequences as paired units; both raw and Holm-adjusted sequence-level p values are reported in Supplementary Table S1. For the sequence-disjoint checkpoint analysis, the five held-out video sequences are treated as the independent evaluation units, and condition-wise sequence-macro DSC is reported as mean ± SD across the five sequences. This analysis is interpreted as a robustness/sensitivity check rather than as a replacement for the original controlled evaluation.

## 5 Results

Following the experimental setup in Section 4, we first compare the overall performance of different test-time adaptation methods across the 12 controlled visual-degradation conditions. We then analyze LiteST-TTA in terms of degradation type and severity, the contribution of temporal constraints, online update behavior, computational cost, and the effect of source-checkpoint selection. A sequence-disjoint held-out analysis is used to test whether the principal degradation-dependent observations persist when checkpoint selection and final evaluation sequences are completely separated. Finally, representative successful and failed adaptation cases are visualized to complement the quantitative analysis.

### 5.1 Overall performance comparison

Table 3 reports the macro-average results of Source, BN Adapt, TENT, Selective Entropy, and LiteST-TTA across the 12 controlled visual-degradation conditions. The macro average assigns equal weight to each test condition and is used to compare overall behavior under diverse controlled degradations.

**TABLE 3:** Macro-average performance across 12 controlled visual-degradation conditions.

Method	DSC ↑	NSD ↑	TC ↑	Update rate	Latency (ms/frame)
Source	0.8533	0.6491	0.3740	0.0%	9.75
BN adapt	0.8864	0.6862	0.3871	0.0%	11.07
TENT	0.8881	0.6918	0.3890	100.0%	41.58
Selective entropy	0.8867	0.6867	0.3875	23.34%	28.10
LiteST-TTA	0.8879	0.6907	0.3887	81.21%	70.70

As shown in Table 3, the fixed Source model obtains macro-average DSC, NSD, and TC values of 0.8533, 0.6491, and 0.3740, respectively. BN Adapt increases these values to 0.8864, 0.6862, and 0.3871 without loss-based gradient optimization, showing that test-time BN-statistics adaptation accounts for a large fraction of the aggregate recovery under the controlled degradations.

The mechanism ablation in Table 4 shows that the statistics-only configuration reaches a macro-average DSC of 0.8864, whereas the joint statistics-plus-affine configuration reaches 0.8879, a difference of 0.001447. In contrast, affine-only optimization with frozen source running statistics falls to 0.8473 overall, mainly because it fails under the strongest low-light shift. Thus, test-time BN-statistics adaptation is the dominant source of aggregate robustness in the present setting, while affine optimization provides only a modest additional macro-average improvement when combined with test-time statistics.

**TABLE 4:** BN mechanism ablation across the 12 controlled degradation conditions.

BN mode	Test-time BN statistics	Gradient optimization of γ,β	Macro DSC	Macro NSD	Macro TC
Statistics only	Updated	No	0.8864	0.6862	0.3872
Affine only	Frozen at source	Yes	0.8473	0.6455	0.3770
Statistics + affine	Updated	Yes	0.8879	0.6907	0.3887

Among the five main methods, TENT achieves the highest macro-average performance, with DSC, NSD, and TC values of 0.8881, 0.6918, and 0.3890, respectively. LiteST-TTA obtains 0.8879, 0.6907, and 0.3887. The macro-average DSC difference between LiteST-TTA and TENT is 0.000281, and the small magnitude of this difference should be interpreted together with the condition-wise dispersion and paired statistical analyses reported below rather than as evidence of a practically meaningful separation.

Under the original uncertainty-gated configurations, LiteST-TTA exceeds Selective Entropy by 0.001167 DSC, 0.003956 NSD, and 0.001176 TC on the macro average and has higher DSC in all 12 degradation cells. These descriptive differences do not, however, isolate the causal contribution of the temporal term because the two adaptive trajectories produce substantially different update rates (23.34% for Selective Entropy and 81.21% for LiteST-TTA). Section 5.3 therefore reports a matched-update control in which both methods update exactly the same frames.

The methods also differ substantially in update frequency and computational cost. TENT updates every test frame, resulting in a 100.0% update rate and an average latency of 41.58 ms/frame. Selective Entropy reduces the update rate to 23.34% through uncertainty gating and lowers latency to 28.10 ms/frame. LiteST-TTA has an average update rate of 81.21%; because it additionally performs motion compensation, temporal pseudo-label construction, and a second forward pass after updating, its average latency increases to 70.70 ms/frame. These values describe the complete original pipelines and emphasize that small differences in segmentation metrics must be interpreted jointly with adaptation frequency and online cost.

To reveal condition-specific behavior beyond the macro averages, Figure 7 presents DSC, NSD, and TC for all five methods under each of the 12 visual degradations. The relative ordering of the methods is not identical across conditions, indicating that the effectiveness of online adaptation depends strongly on the degradation type and severity.

**FIGURE 7:**
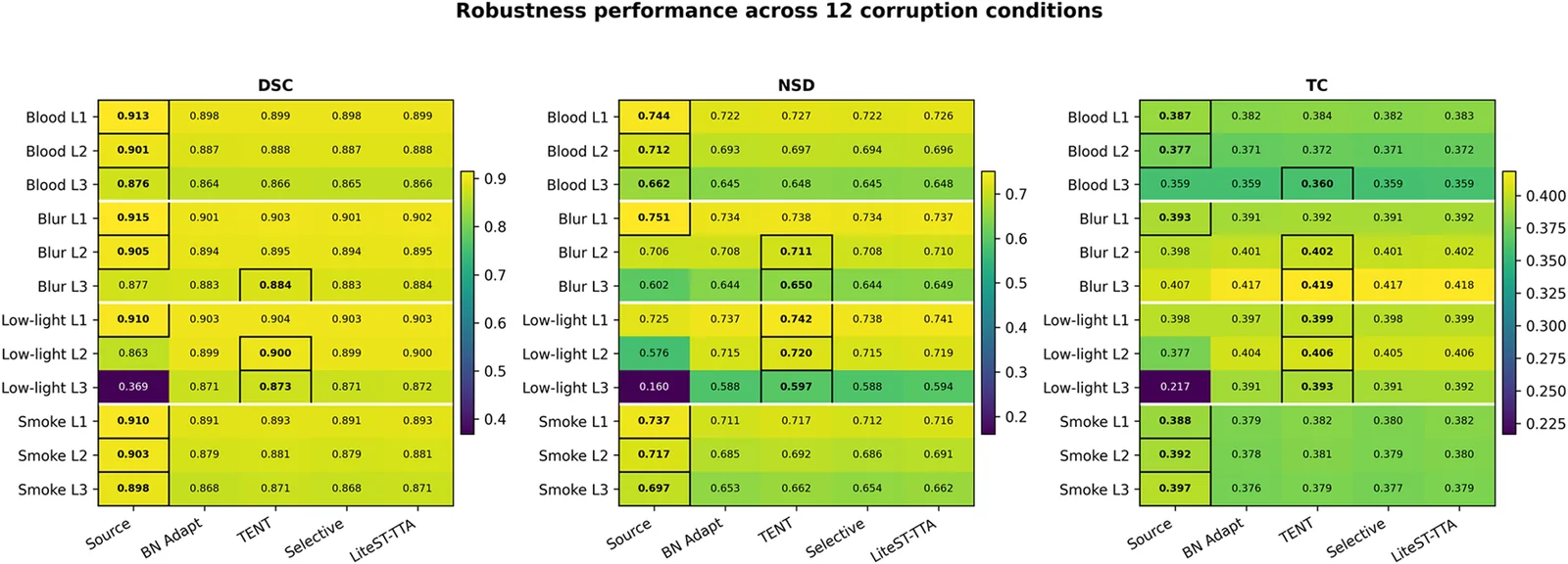
DSC, NSD, and TC of the five methods across 12 controlled visual-degradation conditions.

Figure 7 further shows that the Source model remains competitive under most mild degradations, whereas test-time adaptation becomes more beneficial under some stronger blur and low-light settings. TENT and LiteST-TTA generally remain close across the test conditions. These condition-specific differences show that macro-average performance alone cannot fully characterize adaptation behavior, motivating the more detailed analysis below.

### 5.2 Adaptation characteristics across visual degradations

Table 5 provides the DSC of all methods under each degradation type and severity and reports the difference between LiteST-TTA and Source.

**TABLE 5:** Image-level DSC mean ± SD under 12 controlled visual-degradation conditions (n = 901 frames per condition).

Condition	Source	BN adapt	TENT	Selective	LiteST-TTA	Δ LiteST−Source
Blood L1	0.913 ± 0.102	0.898 ± 0.121	0.899 ± 0.120	0.898 ± 0.120	0.899 ± 0.120	−0.0139
Blood L2	0.901 ± 0.105	0.887 ± 0.123	0.888 ± 0.122	0.887 ± 0.122	0.888 ± 0.122	−0.0130
Blood L3	0.876 ± 0.118	0.864 ± 0.133	0.866 ± 0.132	0.865 ± 0.133	0.866 ± 0.132	−0.0107
Blur L1	0.915 ± 0.100	0.901 ± 0.119	0.903 ± 0.118	0.901 ± 0.119	0.902 ± 0.118	−0.0127
Blur L2	0.905 ± 0.105	0.894 ± 0.125	0.895 ± 0.124	0.894 ± 0.125	0.895 ± 0.124	−0.0104
Blur L3	0.877 ± 0.130	0.883 ± 0.128	0.884 ± 0.126	0.883 ± 0.127	0.884 ± 0.127	+0.0074
Low-light L1	0.910 ± 0.103	0.903 ± 0.119	0.904 ± 0.118	0.903 ± 0.119	0.903 ± 0.118	−0.0061
Low-light L2	0.863 ± 0.143	0.899 ± 0.118	0.900 ± 0.118	0.899 ± 0.118	0.900 ± 0.118	+0.0365
Low-light L3	0.369 ± 0.285	0.871 ± 0.119	0.873 ± 0.119	0.871 ± 0.119	0.872 ± 0.119	+0.5038
Smoke L1	0.910 ± 0.104	0.891 ± 0.126	0.893 ± 0.125	0.891 ± 0.126	0.893 ± 0.125	−0.0170
Smoke L2	0.903 ± 0.108	0.879 ± 0.135	0.881 ± 0.134	0.879 ± 0.135	0.881 ± 0.134	−0.0226
Smoke L3	0.898 ± 0.115	0.868 ± 0.138	0.871 ± 0.137	0.868 ± 0.137	0.871 ± 0.136	−0.0269

**TABLE 6:** Sequence-level repeated-measures ANOVA for LiteST-TTA (n = 10 video sequences).

Outcome	Effect	F (df1, df2)	GG-corrected p	Partial η2
DSC	Degradation type	3.066 (3,27)	0.079	0.254
DSC	Severity	63.630 (2,18)	<0.001	0.876
DSC	Type × severity	4.080 (6,54)	0.031	0.312
ΔDSC vs. source	Degradation type	75.083 (3,27)	<0.001	0.893
ΔDSC vs. source	Severity	67.939 (2,18)	<0.001	0.883
ΔDSC vs. source	Type × severity	74.778 (6,54)	<0.001	0.893

For transparency, the Greenhouse-Geisser epsilon (ε) values were 0.5951, 0.5500, and 0.3549 for degradation type, severity, and their interaction, respectively, in the DSC analysis, and 0.3433, 0.5613, and 0.1975 for the corresponding effects in the ΔDSC analysis. The F statistics and degrees of freedom shown in Table 6 are uncorrected, whereas the reported p values are Greenhouse-Geisser corrected.

Table 5 shows pronounced condition dependence in both the mean DSC and its image-level dispersion. LiteST-TTA exceeds Source in mean DSC under Blur L3, Low-light L2, and Low-light L3, while it is lower in the other 9 cells. Holm-adjusted frame-level Wilcoxon tests classify 3 cells as significantly better than Source, eight as significantly worse, and one as not significant; sequence-level sensitivity tests are used alongside these frame-level results because neighboring video frames are not independent. The presence of visual degradation therefore does not by itself imply that online parameter adjustment will improve the current prediction.

The low-light results exhibit a particularly clear severity-dependent pattern. Under Low-light L1, Source retains a DSC of 0.909543 while LiteST-TTA reaches 0.903464. At L2, Source decreases to 0.863487 whereas LiteST-TTA rises to 0.899986. Under Low-light L3, Source collapses to 0.368579 while LiteST-TTA recovers to 0.872368, an absolute improvement of 0.503789. TENT reaches 0.872819 under the same condition and remains close to LiteST-TTA. Thus, when severe low illumination causes clear source-model failure, online adjustment can effectively recover instrument segmentation.

Blur shows a similar but less pronounced severity-dependent trend. Under Blur L1 and L2, Source obtains DSC values of 0.914951 and 0.905425, both higher than the test-time adaptation methods. When blur increases to L3, Source falls to 0.876619, while TENT and LiteST-TTA reach 0.884367 and 0.884044. The compensatory effect of online adaptation therefore emerges only once blur becomes sufficiently strong to degrade the source model.

Blood and Smoke exhibit a different pattern. Source achieves the highest DSC at all three Blood severity levels, and a similar tendency is observed for Smoke. For Smoke L1, L2, and L3, the DSC differences between LiteST-TTA and Source are −0.016983, −0.022597, and −0.026851, respectively, becoming increasingly negative with severity. Greater degradation severity therefore does not necessarily produce greater adaptation benefit. When the source model still handles the visual change well, additional parameter adjustment can instead impair an already effective prediction.

Figure 8 reorganizes these relative changes into four degradation-specific panels so that L1-L3 are interpreted only within each degradation type rather than as a shared severity scale. Within the Low-light panel, adaptation changes from a slight negative effect at L1 to a positive effect at L2 and a large improvement at L3. Blur shows a smaller transition, with positive changes emerging only at L3, whereas Blood and Smoke remain predominantly negative across their within-type severity levels. The four-panel presentation therefore emphasizes that adaptation benefit is jointly conditioned by degradation type and within-type severity, not by a globally comparable severity index.

**FIGURE 8:**
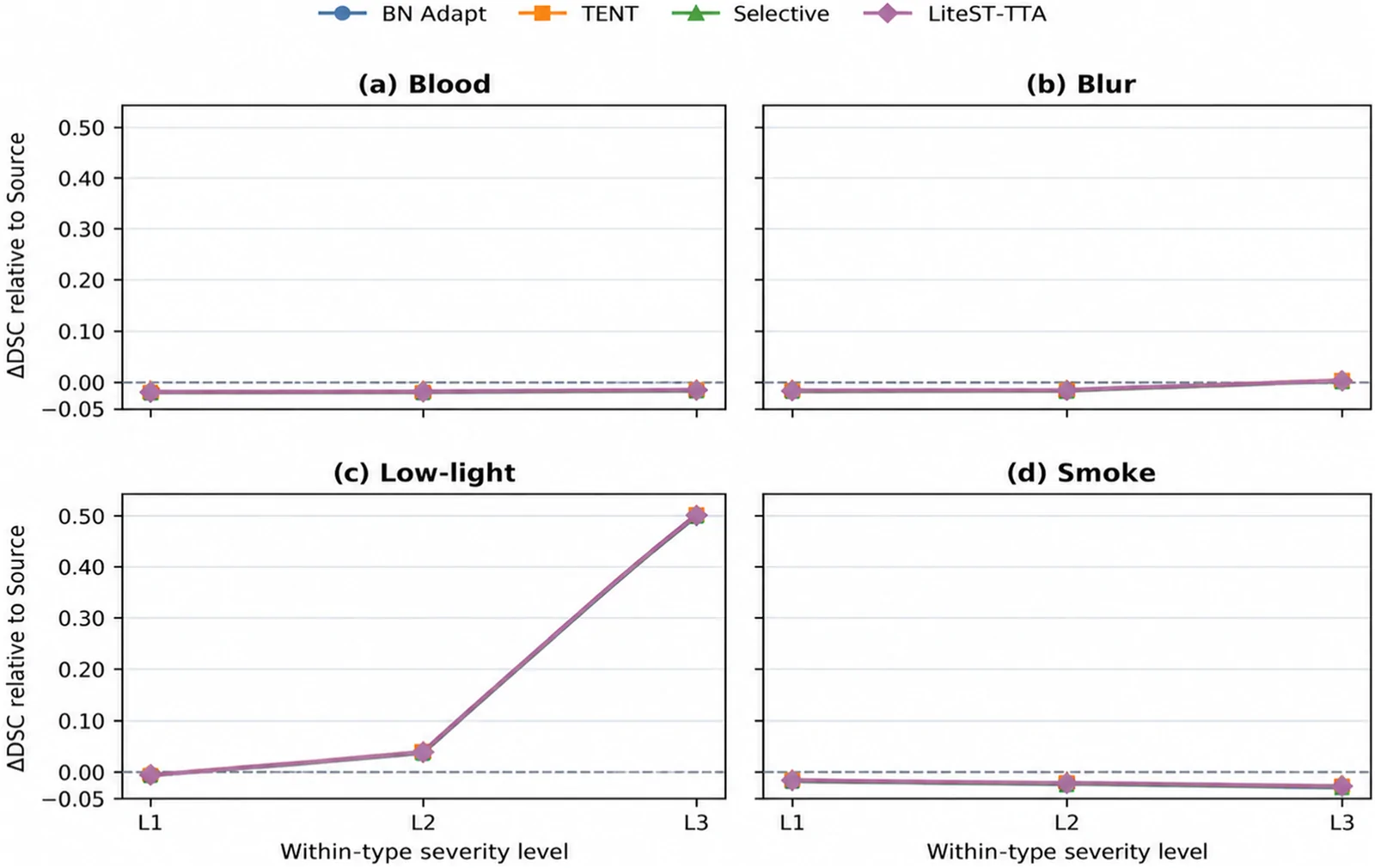
DSC changes relative to Source across four degradation types: **(a)** Blood, **(b)** Blur, **(c)** Low-light, and **(d)** Smoke. L1-L3 are degradation-specific ordinal levels and are not directly comparable across types; positive and negative values indicate improvement and degradation relative to Source, respectively.

Macro averages can also be strongly affected by a small number of extreme conditions. Figure 9 therefore shows the distribution of DSC across all 12 conditions for each method together with the corresponding macro-average level.

**FIGURE 9:**
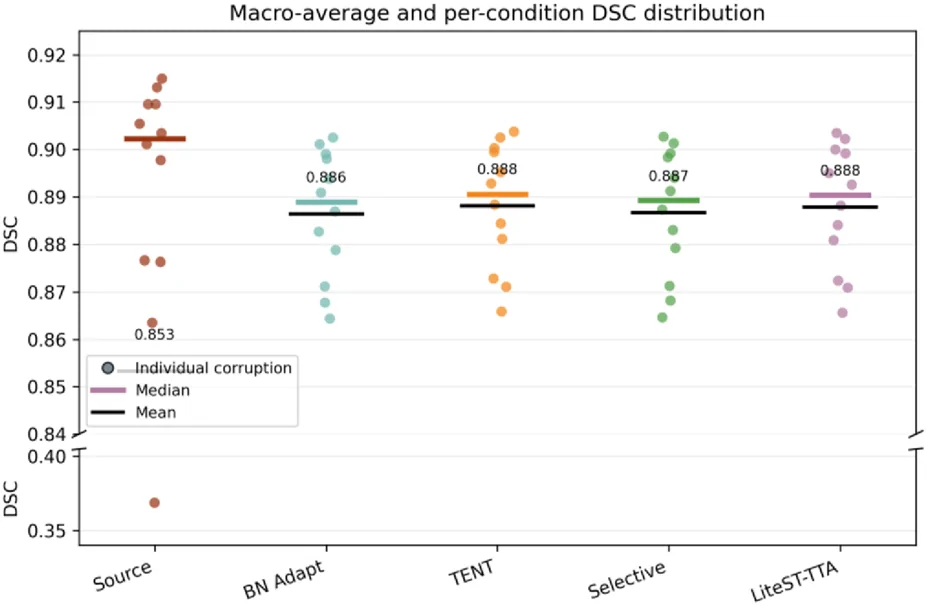
Distribution of DSC across the 12 controlled visual-degradation conditions.

As shown in Figure 9, Source remains concentrated in a high DSC range for most conditions, whereas its value of 0.368579 under Low-light L3 is a pronounced outlier. From a descriptive-statistics perspective, if this extreme condition is temporarily excluded, Source averages approximately 0.8974 across the remaining 11 conditions, compared with approximately 0.8893 for LiteST-TTA. Thus, the lower macro-average value of Source in Table 3 is driven largely by the severe low-light failure rather than by uniformly inferior performance across all degradations.

To formally test the degradation-dependence claim, we performed a two-way repeated-measures ANOVA at the video-sequence level (n = 10). For absolute LiteST-TTA DSC, severity had a strong main effect (F (2,18) = 63.630, Greenhouse-Geisser corrected p < 0.001, partial eta squared = 0.876), while the type-by-severity interaction was also significant (F (6,54) = 4.080, corrected p = 0.031, partial eta squared = 0.312); the corrected main effect of degradation type did not reach 0.05 (F (3,27) = 3.066, p = 0.079). More directly for adaptation benefit, when ΔDSC relative to Source was used as the response, degradation type, severity, and their interaction were all significant after Greenhouse-Geisser correction (all p < 0.001), with partial eta squared values of 0.893, 0.883, and 0.893, respectively. These results support a degradation-type-by-severity interaction in adaptation benefit rather than an interpretation based only on the visual ordering of the 12 cells.

Full condition-wise paired statistics for LiteST-TTA *versus* Source, BN Adapt, TENT, and Selective Entropy are reported in Supplementary Table S1, including the paired mean DSC difference, 95% confidence interval, Cohen’s dz, frame-level Wilcoxon p value, Holm-adjusted frame-level p value, paired t-test p value, and both raw and Holm-adjusted sequence-level Wilcoxon p values. At the frame level, LiteST-TTA is significantly better than Source in three of 12 conditions, significantly worse in 8, and not significant in 1; it is higher than BN Adapt and Selective Entropy in all 12 cells and lower than TENT in all 12 cells. After Holm correction of the sequence-level sensitivity tests across the 12 cells, four LiteST-TTA-versus-Source comparisons remain below 0.05 (Low-light L3 in the positive direction and Smoke L1-L3 in the negative direction), five comparisons *versus* BN Adapt remain below 0.05, two *versus* Selective Entropy remain below 0.05, and none *versus* TENT remain below 0.05. This contrast illustrates why very small frame-level effects should not be interpreted as equally strong sequence-level evidence.

### 5.3 Temporal constraints, matched-update control, and component ablation

Selective Entropy and LiteST-TTA both use predictive uncertainty to trigger online adaptation, but the two methods evolve different model states after an update and therefore do not preserve an identical gate trajectory. In the original configurations, LiteST-TTA has higher DSC than Selective Entropy in all 12 degradation cells, yet its update rate is 81.21% compared with 23.34% for Selective Entropy. The original 0.001167 macro-average DSC difference therefore combines the effect of the temporal objective with the effect of a substantially different update trajectory and cannot be attributed to temporal information alone.

To isolate this issue, we forced both methods to follow the same periodic update schedule: the first frame of each video sequence was not updated and every fourth subsequent frame was updated. This produced the same macro-average update rate of 24.20% and, crucially, the same update-frame identities for both methods. Under this matched budget, Selective Entropy achieved a macro-average DSC of 0.886966 and LiteST-TTA achieved 0.886967. Across all 10,812 paired frame evaluations, the mean DSC difference was 0.0000006 with a 95% confidence interval of [-0.000002, 0.000003] and Cohen’s dz = 0.0044; the sequence-level Wilcoxon test was not significant (p = 0.496). Although the frame-level rank test was significant because of the very large paired sample, the effect size was effectively zero and the confidence interval crossed zero. Thus, under an equal update budget there is no evidence of a meaningful standalone DSC advantage from the temporal term. The matched-update comparison between Selective Entropy and LiteST-TTA is summarized in Table 7.

**TABLE 7:** Matched-update comparison between Selective Entropy and LiteST-TTA.

Method	Update schedule	Macro update rate	Macro DSC
Selective entropy	First frame skipped; then every 4th frame	24.20%	0.886966
LiteST-TTA	First frame skipped; then every 4th frame	24.20%	0.886967

The matched-update result changes the interpretation of the original comparison. Temporal regularization remains part of the complete LiteST-TTA, trajectory and can alter future entropy values, update decisions, boundary behavior, and temporal smoothness, but the descriptive DSC, gain observed under the original gate should not be presented as an independently identified causal effect of the temporal-consistency term.

To determine how the temporal supervision design and rollback mechanism affect adaptation, we use Selective Entropy as the reference baseline and perform component ablations for several LiteST-TTA variants. The results are shown in Figure 10.

**FIGURE 10:**
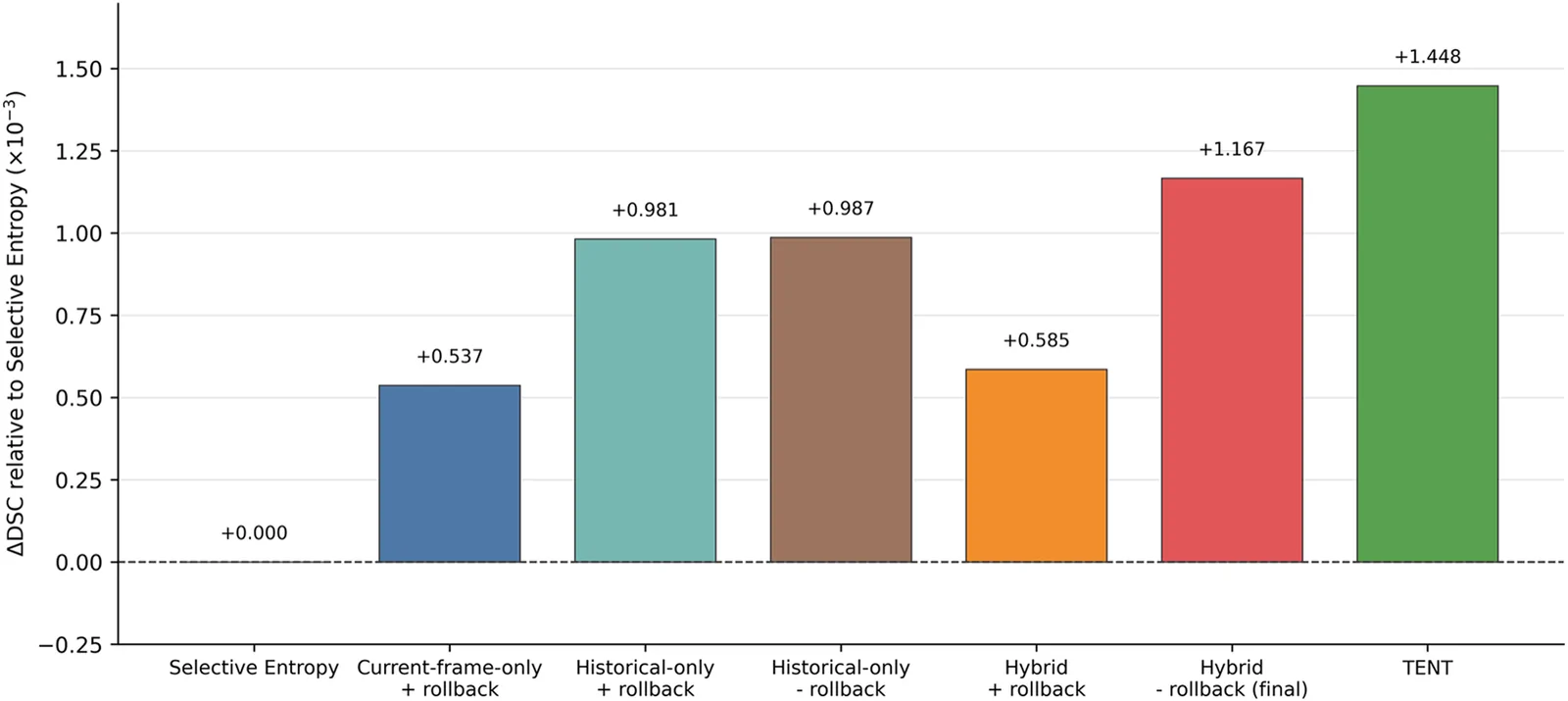
Unified component ablation under the final 12-condition protocol. Bars show macro-average ΔDSC relative to Selective Entropy (×10^–3^); TENT is included as an external reference. All ablation variants use the same source checkpoint, 12 controlled degradation conditions, and final test-time parameter settings.

Under the unified final protocol, the macro-average DSC values are 0.886690 for Selective Entropy, 0.887227 for current-frame-only + rollback, 0.887672 for historical-only + rollback, 0.887677 for historical-only without rollback, 0.887276 for hybrid + rollback, 0.887857 for the final hybrid configuration without rollback, and 0.888138 for TENT. The requested historical-only configuration without rollback improves on Selective Entropy by +0.000987. Removing rollback from historical-only changes macro-average DSC by only +0.000005 (95% CI [−0.000015, +0.000026]; sequence-level Wilcoxon p = 0.496), showing that the rollback effect observed for the hybrid mask is not additive across temporal-mask designs. Historical-only without rollback remains 0.000180 below the final hybrid configuration and 0.000461 below TENT. The final rollback-disabled hybrid therefore remains the highest observed LiteST-TTA variant, while TENT remains slightly higher as an external reference; all of these differences are small in absolute terms.

To directly assess the rollback choice, we performed a paired comparison between the final hybrid configuration without rollback and the otherwise matched hybrid configuration with rollback. Across all 10,812 paired frame evaluations, disabling rollback increases DSC by +0.000581 on average (95% CI [+0.000548, +0.000615], Cohen’s dz = 0.3278), and the aggregate sequence-level Wilcoxon test across the 10 video sequences gives p = 0.0273. The mean difference is positive in all 12 degradation cells; however, after Holm correction of the 12 condition-wise sequence-level tests, only Smoke L2 remains below 0.05 (adjusted p = 0.0469). The rollback-disabled setting is therefore retained because it gives the highest observed aggregate DSC under the unified protocol, but the effect is described as small and its condition-wise stability is not overstated. Full rollback on/off paired statistics are provided in Supplementary Table S3.

### 5.4 Online adaptation behavior and computational efficiency

Test-time adaptation changes not only segmentation quality but also the frequency of online parameter updates and the per-frame processing time. Figure 11 compares the methods in terms of macro-average DSC, update rate, and average latency: the horizontal axis represents latency, the vertical axis represents macro-average DSC, and bubble size encodes update rate.

**FIGURE 11:**
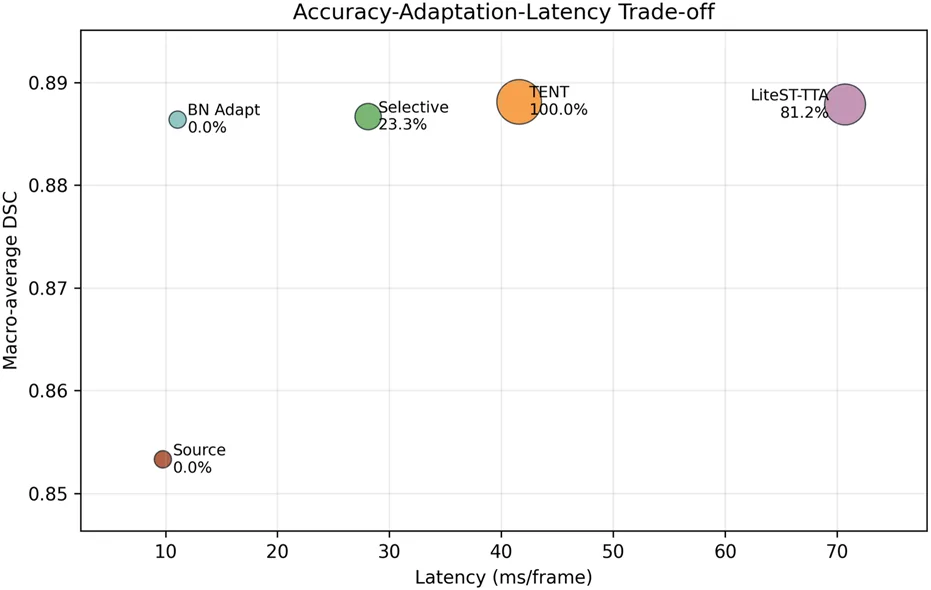
Performance-adaptation-latency trade-off. The x-axis denotes average latency, the y-axis denotes macro-average DSC, and bubble size represents the parameter-update rate.

TENT updates every test frame, giving an update rate of 100.0%, a macro-average DSC of 0.8881, and an average observed computation/adaptation latency of 41.58 ms/frame. Selective Entropy reduces the update rate to 23.34% using uncertainty gating, lowering latency to 28.10 ms/frame while obtaining a macro-average DSC of 0.8867. LiteST-TTA has an update rate of 81.21% and a macro-average DSC of 0.8879, but its additional optical-flow estimation, temporal-supervision construction, gradient update, and final forward pass increase latency to 70.70 ms/frame. Under the common timing implementation, TENT and LiteST-TTA correspond to approximately 24.1 and 14.1 frames/s, respectively. Because the matched-update analysis finds virtually identical DSC for Selective Entropy and LiteST-TTA when they update the same frames, the higher original LiteST-TTA score should be interpreted jointly with its different adaptive trajectory and higher computational cost rather than as an isolated temporal gain.

Beyond update frequency and latency, an important question for selective adaptation is whether predictive uncertainty can indicate the actual effectiveness of an online update. Figure 12 compares the average predictive uncertainty, update rate, and DSC change relative to Source for LiteST-TTA across degradation types and severity levels.

**FIGURE 12:**
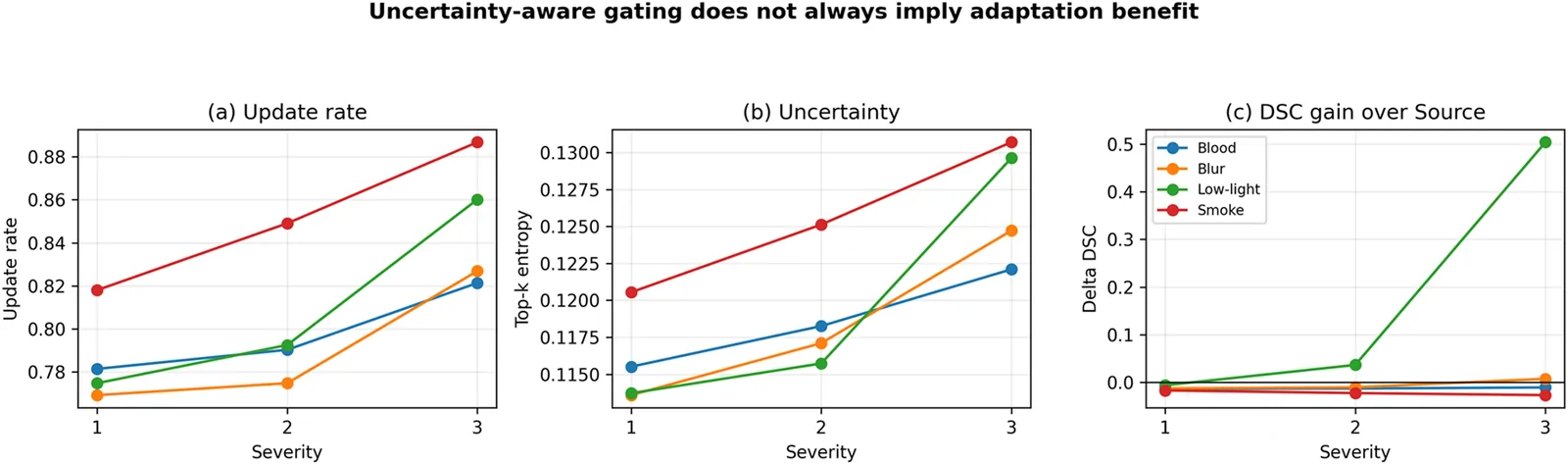
Effects of degradation severity on **(a)** update rate, **(b)** predictive uncertainty, and **(c)** DSC gain relative to Source for Blood, Blur, Low-light, and Smoke.

Figure 12 shows that predictive uncertainty and update rate generally increase from L1 to L3 for most degradation types, indicating that the Top- k entropy gate responds to changes in the model’s prediction state. However, these increases do not consistently correspond to improved DSC after adaptation. Under Smoke, for example, LiteST-TTA maintains a high update frequency as severity increases, yet its DSC differences relative to Source remain negative at −0.016983, −0.022597, and −0.026851. In contrast, Low-light L2 and L3 combine high uncertainty with clear positive DSC changes. Predictive uncertainty therefore reflects the confidence state of the current output but cannot by itself determine whether a parameter update will improve segmentation.

To further examine these mechanisms over continuous video, Figure 13 shows frame-wise DSC, predictive uncertainty, and update events for a representative sequence under severe low illumination.

**FIGURE 13:**
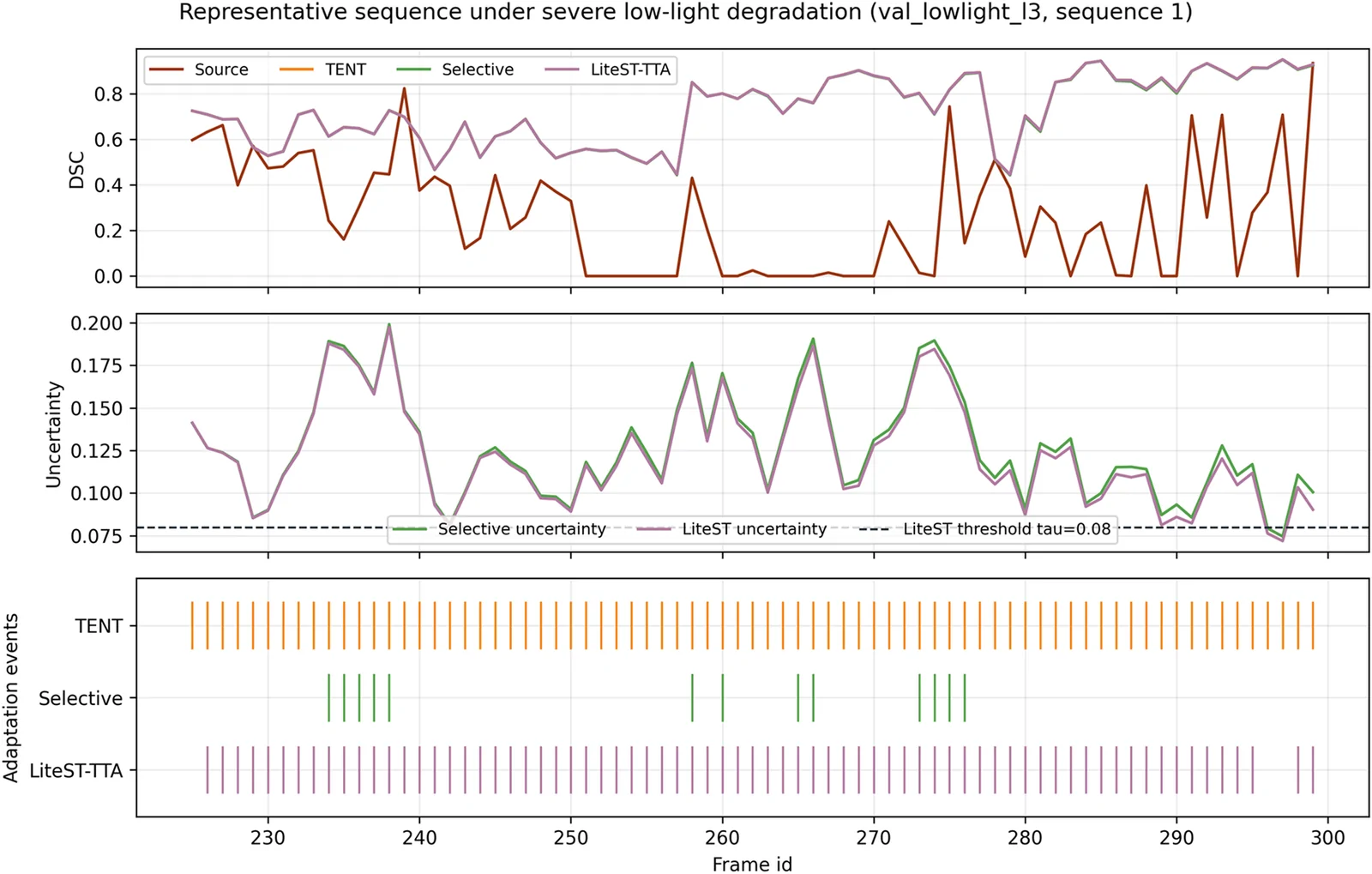
Frame-wise DSC, predictive uncertainty, and online update events in a representative severe low-light sequence.

Figure 13 shows that Source undergoes pronounced performance degradation over part of the sequence, whereas the adaptive methods maintain higher DSC over the corresponding interval. TENT follows its continual-adaptation strategy and updates throughout the sequence, while Selective Entropy and LiteST-TTA use uncertainty-dependent updating. Although the two selective methods share the same initial gate definition and threshold, their model states can diverge after the first different optimization step; subsequent predictive-entropy trajectories, and therefore later update decisions, are then state dependent. The gate should thus not be treated as an independent control variable across the two adaptive methods once online adaptation has begun.

Taken together, Figures 10–12 and the matched-update control show that online adaptation performance cannot be explained by update frequency or predictive uncertainty alone. Continual updating can achieve strong aggregate performance, uncertainty gating can reduce some online operations, and temporal regularization changes the adaptive trajectory, but the matched-update analysis does not support a meaningful standalone DSC benefit from the temporal term under an equal update budget. Explicit motion compensation also increases processing cost. Moreover, high predictive uncertainty does not necessarily correspond to beneficial parameter adjustment, a finding examined further in the Discussion.

### 5.5 Sequence-disjoint checkpoint-selection sensitivity analysis

The sequence-disjoint analysis directly tests whether the original degradation-dependent findings can be explained solely by selecting the Source checkpoint on the same underlying validation content used for degraded evaluation. Using only the five checkpoint-selection sequences, epoch 38 is selected from the complete set of 40 saved checkpoints (clean frame-mean DSC = 0.952862; sequence-macro DSC = 0.950520). This checkpoint is then frozen before any evaluation is performed on the five held-out sequences. The clean frame-mean DSC of 0.952862 is reported only to document checkpoint selection within the 301-frame subset and is not directly comparable with the 0.9168 value used to select epoch 34 in the original protocol, because the latter was computed over all 901 validation frames from 10 sequences. The two values use different evaluation subsets and therefore do not indicate that epoch 38 is intrinsically better than epoch 34. The sequence-macro DSC results across the 12 degradation conditions and five held-out video sequences are presented in Table 8.

**TABLE 8:** Sequence-disjoint sensitivity analysis: sequence-macro DSC mean ± SD across five held-out video sequences.

Condition	Source	BN adapt	TENT	Selective entropy	LiteST-TTA
Blood L1	0.8639 ± 0.0958	0.8353 ± 0.0807	0.8375 ± 0.0817	0.8361 ± 0.0808	0.8372 ± 0.0814
Blood L2	0.8464 ± 0.0920	0.8205 ± 0.0813	0.8231 ± 0.0811	0.8213 ± 0.0812	0.8228 ± 0.0808
Blood L3	0.8163 ± 0.0947	0.7945 ± 0.0870	0.7966 ± 0.0865	0.7952 ± 0.0866	0.7964 ± 0.0862
Blur L1	0.8664 ± 0.0967	0.8412 ± 0.0834	0.8440 ± 0.0841	0.8422 ± 0.0835	0.8437 ± 0.0837
Blur L2	0.8563 ± 0.0949	0.8292 ± 0.0885	0.8322 ± 0.0879	0.8303 ± 0.0879	0.8320 ± 0.0875
Blur L3	0.8262 ± 0.0940	0.8166 ± 0.0854	0.8194 ± 0.0861	0.8178 ± 0.0853	0.8191 ± 0.0856
Low-light L1	0.8500 ± 0.0859	0.8430 ± 0.0830	0.8452 ± 0.0846	0.8436 ± 0.0833	0.8448 ± 0.0841
Low-light L2	0.7790 ± 0.0911	0.8386 ± 0.0794	0.8407 ± 0.0806	0.8392 ± 0.0795	0.8403 ± 0.0802
Low-light L3	0.3251 ± 0.1929	0.8062 ± 0.0679	0.8087 ± 0.0690	0.8070 ± 0.0677	0.8083 ± 0.0686
Smoke L1	0.8568 ± 0.1024	0.8255 ± 0.0885	0.8278 ± 0.0899	0.8261 ± 0.0886	0.8275 ± 0.0896
Smoke L2	0.8489 ± 0.0994	0.8110 ± 0.0888	0.8137 ± 0.0901	0.8116 ± 0.0889	0.8133 ± 0.0897
Smoke L3	0.8297 ± 0.0948	0.7971 ± 0.0939	0.8006 ± 0.0955	0.7983 ± 0.0939	0.8004 ± 0.0951
Macro average	0.7971 ± 0.0853	0.8216 ± 0.0833	0.8241 ± 0.0841	0.8224 ± 0.0833	0.8238 ± 0.0837

On the completely held-out sequences, the dominant low-light pattern remains. LiteST-TTA, exceeds Source in sequence-macro DSC, under Low-light L2 (0.8403 ± 0.0802 *versus*0.7790 ± 0.0911; Δ = +0.061330) and Low-light L3 (0.8083 ± 0.0686 *versus*0.3251 ± 0.1929; Δ = +0.483148), while Source remains higher in the other 10 of 12 degradation cells. Under Low-light L3, BN, adapt, Selective Entropy; LiteST-TTA, and TENT, reach 0.8062 ± 0.0679, 0.8070 ± 0.0677, 0.8083 ± 0.0686, and 0.8087 ± 0.0690, respectively, compared with 0.3251 ± 0.1929 for Source. Across the five held-out sequences, the 12-condition macro averages are 0.7971 ± 0.0853 for Source, 0.8216 ± 0.0833 for BN, adapt, 0.8241 ± 0.0841 for TENT, 0.8224 ± 0.0833 for Selective Entropy, and 0.8238 ± 0.0837 for LiteST-TTA., Reporting between-sequence variability makes clear that the severe low-light recovery is large relative to the observed sequence-to-sequence spread, while the much smaller differences among the adaptive methods should be interpreted cautiously.

The held-out analysis therefore supports the broad conclusion that adaptation is most useful when low illumination causes a clear source-model failure and that unnecessary adaptation can remain detrimental when the source model is already robust. At the same time, not every individual cell-level effect is invariant to the sequence split: the positive LiteST-TTA change observed for Blur L3 in the original 901-frame evaluation becomes a small negative change in the held-out analysis (Δ = −0.007040). The sequence-disjoint experiment should therefore be interpreted as evidence that the principal degradation-dependent pattern is not solely an artifact of checkpoint-selection overlap, rather than as proof that every original condition-specific difference generalizes unchanged.

### 5.6 Qualitative analysis

To visualize how test-time adaptation changes the segmented instrument region, we select representative cases from different visual degradations. The qualitative comparison includes both conditions in which adaptation improves the source prediction and conditions in which performance declines after adaptation, avoiding an evaluation based only on favorable examples. The results are shown in Figure 14.

**FIGURE 14:**
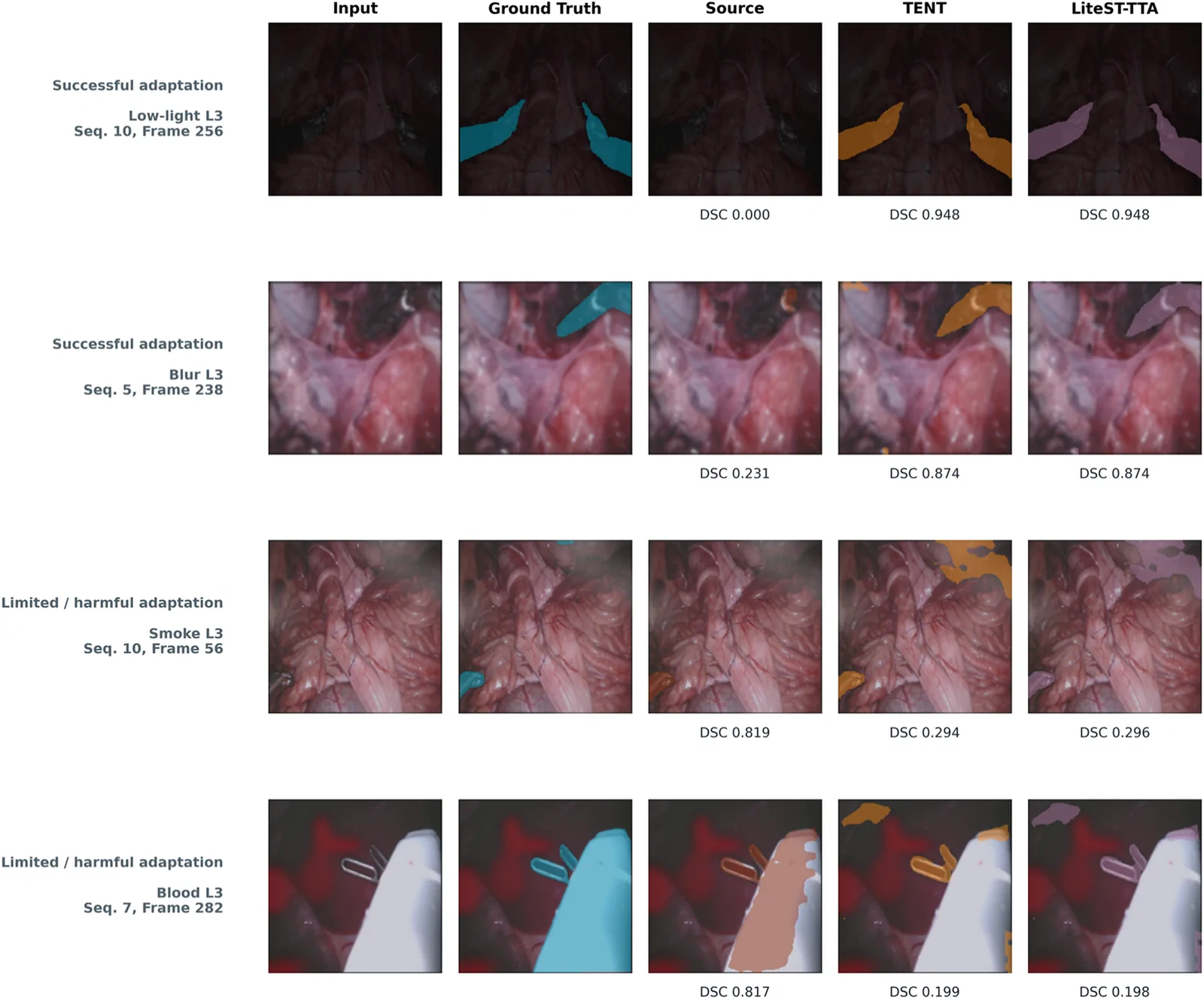
Qualitative segmentation comparison under different visual degradations. Columns show Input, Ground Truth, Source, TENT, and LiteST-TTA; the first two rows illustrate successful adaptation under severe low-light and blur, whereas the last two rows illustrate limited or harmful adaptation under Smoke and Blood.

In the first two rows of Figure 14, the source model exhibits clear degradation under severe low illumination and strong blur. In the Low-light L3 example, Source fails to segment the instrument and obtains a DSC of 0, whereas both TENT and LiteST-TTA recover to approximately 0.948. In the Blur L3 example, Source obtains a DSC of only 0.231, while TENT and LiteST-TTA reach approximately 0.875 and 0.874, respectively. These examples show that online parameter adjustment can recover a more complete instrument region when visual degradation has substantially disrupted the source model’s representation.

The last two rows of Figure 14 show a different behavior. In representative Smoke L3 and Blood L3 samples, Source obtains DSC values of approximately 0.819 and 0.817, indicating that the fixed source model still segments the instrument well. After test-time adaptation, the predicted regions deviate markedly and DSC decreases to approximately 0.29 and 0.20. This behavior is consistent with the condition-level trends in Table 5 and Figure 8 and illustrates that additional unsupervised parameter adjustment can damage an already effective source prediction.

The quantitative and qualitative analyses therefore lead to the same conclusion: the effect of TTA is strongly scenario dependent. Online adaptation can recover segmentation when severe low illumination or strong blur causes clear source-model failure, whereas continuing to adapt under some Blood and Smoke conditions can reduce performance because the source model is already in a relatively robust operating regime. The decision to update at test time therefore cannot be based solely on the presence of visual degradation or on predictive uncertainty.

Across the original main comparison, TENT achieves the highest macro-average performance, while LiteST-TTA remains close in DSC, NSD, and TC. Sequence-level factorial analysis confirms that adaptation benefit depends on the interaction between degradation type and severity. The original LiteST-TTA configuration is higher than Selective Entropy in all 12 cells, but the matched-update experiment shows that this difference disappears when both methods update the same frames, limiting causal attribution to the temporal term alone. The BN mechanism ablation further shows that test-time normalization statistics explain most of the aggregate recovery. Together, these observations indicate that degradation severity, source-model robustness, normalization state, update dynamics, temporal regularization, and computational cost must be considered jointly when interpreting surgical TTA.

## 6 Discussion and future research directions

The preceding experiments show that test-time adaptation in complex intraoperative visual environments is strongly scenario dependent. Its practical behavior is influenced not only by degradation type and severity but also by the current state of the source model, the online update strategy, and the reliability of temporal information. Temporal constraints can provide additional information for unlabeled online optimization, but they also introduce motion-estimation and historical-state overhead. This section therefore discusses the applicability boundary of online adaptation, the limitations of uncertainty gating, the role of temporal and normalization adaptation, and directions toward more realistic and reliable robotic surgical vision.

### 6.1 Applicability boundary of online adaptation under different visual degradations

The experiments show that TTA does not provide uniform improvement across visual degradations. LiteST-TTA produces positive mean DSC changes relative to Source mainly under Blur L3, Low-light L2, and Low-light L3 in the original 901-frame evaluation, with the most pronounced recovery under severe low illumination. Sequence-level repeated-measures analysis supports this descriptive pattern: the type-by-severity interaction is significant for absolute DSC (corrected p = 0.031) and especially strong for ΔDSC relative to Source (corrected p < 0.001). The sequence-disjoint sensitivity analysis further shows that the large Low-light L2/L3 benefits persist when checkpoint selection and evaluation sequences do not overlap, while Source remains higher in 10 of the 12 held-out cells. Blur L3 does not retain a positive LiteST-TTA delta in the held-out split, underscoring that individual cell-level effects should be interpreted more cautiously than the dominant low-light pattern.

This divergence indicates that a visual appearance change does not necessarily mean that the source model has lost effective perception. For test samples that remain within the model’s robustness range, additional unsupervised updating can alter feature statistics or decision boundaries and disturb an otherwise stable prediction. Online adaptation becomes more likely to provide useful compensation only after the degradation has caused clear source-model failure. Under Low-light L3, for example, Source falls to a DSC of 0.368579 whereas LiteST-TTA recovers to 0.872368, illustrating the potential value of TTA when the visual shift meaningfully impairs the deployed model.

For robotic surgical vision, TTA should therefore not be reduced to a rule of “detect distribution change and update.” A more relevant question is whether the current visual change has pushed the source model outside its effective operating range. Future online-adaptation mechanisms could combine current prediction reliability, short-term temporal stability, and historical adaptation states to estimate whether the model truly requires adjustment, thereby reducing unnecessary parameter changes and concentrating online optimization on periods of genuine perception degradation.

### 6.2 Limitations of uncertainty gating and adaptation-effectiveness assessment

Predictive uncertainty describes the current output state of a model and is therefore often used to select samples for test-time updating. LiteST-TTA uses Top- k predictive entropy to construct frame-level uncertainty and control online adaptation. The experiments show that uncertainty and update frequency generally increase as some degradations become more severe, indicating that the measure responds to changes in model confidence during testing.

Higher uncertainty, however, does not necessarily correspond to a more effective update. Under Smoke, the update frequency remains high as degradation severity increases, yet the DSC change of LiteST-TTA relative to Source remains negative and decreases from −0.016983 to −0.026851. Severe low-light conditions, by contrast, combine high uncertainty with substantial performance recovery. Predictive entropy therefore indicates how certain the model is about its current output, but it does not directly indicate whether a subsequent parameter update will improve the prediction.

One reason is that an unlabeled optimization objective can increase confidence without necessarily moving the prediction toward the ground truth. Entropy minimization encourages more decisive outputs but cannot guarantee that the direction of the update is semantically correct; if the current prediction contains a systematic error, further entropy minimization may reinforce the wrong decision. In addition, the matched-update experiment shows that the uncertainty gate is coupled to the evolving model state: different optimization objectives can change subsequent entropy values and therefore change later update frequency. A single threshold is consequently not an independent control variable across adaptive methods and is insufficient to characterize the practical effectiveness of an online update.

Future work could explicitly separate the questions “Is the current prediction unstable?” and “Is a parameter update likely to improve the model state?” Adaptation-effectiveness-aware criteria could incorporate short-term changes in temporal consistency, historical update outcomes, model calibration, or lightweight probe updates in addition to uncertainty. Compared with a gate based solely on $U_{t}>\tau_{u}$ , such mechanisms may reduce updates on high-uncertainty frames for which adaptation is unlikely to be beneficial and thereby improve the reliability of selective TTA in continuous surgical video.

### 6.3 Role of temporal constraints and computational efficiency

Adjacent frames in surgical video exhibit strong structural continuity, allowing historical predictions to provide information unavailable to a purely single-frame objective. In the original uncertainty-gated configurations, LiteST-TTA is higher than Selective Entropy in DSC across all 12 cells and also has higher macro-average NSD and TC. However, these two methods evolve substantially different update rates. When update count and update-frame identity are matched, their macro-average DSC values become virtually identical (0.886967 *versus* 0.886966; sequence-level Wilcoxon p = 0.496; Cohen’s dz = 0.0044). The original difference therefore cannot be attributed solely to the temporal-consistency term; instead, temporal regularization interacts with the evolving model state and subsequent gating trajectory.

The interpretation of TC also requires care. LiteST-TTA explicitly minimizes a motion-compensated temporal-consistency loss, while TC is computed from agreement between motion-compensated consecutive predictions. TC is therefore an optimization-related diagnostic and not independent validation of the temporal mechanism. As an additional flow-independent stability measure, the macro-average absolute frame-to-frame change in ground-truth DSC is 0.03921 for LiteST-TTA, compared with 0.03972 for Selective Entropy, 0.03985 for BN Adapt, 0.05086 for Source, and 0.03908 for TENT. LiteST-TTA thus shows slightly lower frame-to-frame accuracy variation than Selective Entropy, but TENT remains marginally lower on this independent measure. Condition-wise mean ± SD values for this metric are reported in Supplementary Table S2.

Computational cost is another important limitation of the current temporal implementation. Under the common synchronized timing protocol on the RTX 4060 Laptop GPU, LiteST-TTA has an observed average latency of 70.70 ms/frame, higher than TENT and Selective Entropy because of dense optical-flow estimation, temporal-supervision construction, gradient-based updating, and the final post-update forward pass. Relative to BN Adapt at 11.07 ms/frame, this is approximately 6.39× higher latency for an aggregate DSC increment of only 0.001447 (0.145 percentage points). Given that the matched-update DSC difference from Selective Entropy is negligible, the current optical-flow-based temporal implementation should be viewed as a mechanism exploration rather than an efficiency-optimal deployment solution.

Future temporal modules for robotic surgical vision should therefore balance information content, update utility, and efficiency. Potential directions include temporal modeling without explicit dense optical flow, such as short-term feature memory, sparse correspondence, low-resolution motion estimation, or correlations between intermediate features. More importantly, future evaluations should control update budgets when attributing gains to a temporal objective and should use stability metrics that are independent of the optimized temporal loss.

### 6.4 Role of normalization adaptation and mechanism attribution

A further finding is that test-time BN-statistics adaptation produces most of the aggregate improvement. In the statistics-only control, BN layers operate in training mode under no-gradient inference, so current test-batch statistics are used for normalization and the running state is updated, without optimizing γ or β. This configuration reaches a macro-average DSC of 0.886411. In the affine-only control, BN layers remain in evaluation mode, the source running mean and variance are frozen and used during the forward pass, and only γ and β are optimized; its macro-average DSC is 0.847343. The joint statistics-plus-affine configuration reaches 0.887858. The +0.001447 increment of the joint configuration over statistics-only adaptation is therefore modest, whereas freezing the source BN statistics and optimizing affine parameters alone is insufficient under the strongest low-light shift. These controls identify test-time normalization-statistics behavior as the dominant aggregate robustness mechanism under the controlled degradations considered here.

This decomposition also changes how the complete LiteST-TTA pipeline should be interpreted. Comparisons against a fixed Source model alone substantially overstate the amount of recovery attributable to entropy minimization, gating, or temporal regularization because the statistics-only BN control already raises DSC from 0.853320 to 0.886411. The additional online machinery provides a modest +0.001447 absolute DSC increment (0.145 percentage points) on top of this strong normalization baseline, while observed latency increases from 11.07 ms/frame for BN Adapt to 70.70 ms/frame for LiteST-TTA, approximately 6.39×. The present results therefore do not establish a favorable deployment cost-benefit trade-off for the additional optical-flow-based temporal machinery; its value should instead be interpreted in terms of mechanism analysis and condition-specific robustness.

The ablation further shows that BN statistics and affine optimization are not interchangeable. Affine-only optimization can remain competitive in several mild or moderate cells, but it becomes unstable under severe domain shift, especially Low-light L3; the configurations using test-time BN statistics do not exhibit the same collapse and provide the primary cross-condition robustness. Joint statistics-plus-affine adaptation yields the highest observed value among the three BN mechanism settings, but its incremental macro-average gain over statistics-only adaptation is small. This result narrows the mechanism attribution of LiteST-TTA and motivates future work on lower-cost normalization-centered adaptation before adding more complex online objectives.

### 6.5 From controlled visual degradation to real continual domain shift

The original controlled evaluation contains source-checkpoint selection overlap: epoch 34 was selected by maximizing DSC on the same clean validation content from which the 12 degraded evaluation conditions were later generated. Although these validation images were not used for gradient-based source-model training, checkpoint selection over a fixed validation set is itself a model-selection step and may favor the Source checkpoint on the underlying scene content. This issue is therefore explicitly separated from the main 901-frame results rather than being treated as ordinary training-data leakage.

To assess the magnitude of this dependence, the sequence-disjoint sensitivity analysis uses five complete video sequences exclusively for checkpoint selection and five different sequences exclusively for final degradation evaluation. With epoch 38 selected only from the checkpoint-selection sequences, the dominant pattern remains: all adaptive methods strongly outperform Source under Low-light L3, and LiteST-TTA is higher than Source under Low-light L2 and L3 but lower in the other 10 held-out cells. This result indicates that the central conclusion about strongly condition-dependent adaptation is not solely explained by reuse of the original validation content for checkpoint selection. However, the analysis uses one fixed 5/5 sequence split and only five held-out sequences, so it is best interpreted as a sensitivity check rather than a substitute for external cross-dataset validation.

The controlled visual-degradation protocol allows different methods to be compared using identical underlying image content and makes it possible to isolate the effects of degradation type and severity. However, this setting primarily represents synthetically constructed visual changes and cannot fully capture the complex domain shifts produced in real surgery by differences in imaging devices, surgical procedures, tissue states, and data-acquisition conditions.

The conclusions of this study should therefore be interpreted within the scope of controlled visual degradation. Future work should incorporate cross-dataset and real cross-domain evaluation. For example, a model trained with EndoVis2017 as the source domain could be adapted directly to other public surgical videos without using target-domain labels for supervised optimization. Such experiments would provide a stronger test of whether the degradation dependence, limitations of uncertainty gating, and role of temporal constraints observed here persist under naturally occurring distribution shifts.

Real surgical deployment also involves continuing changes in illumination, instrument state, and tissue environment over the course of an operation. Our current sequence-level reset strategy limits the propagation of erroneous adaptation across independent video sequences but also discards potentially useful knowledge accumulated during longer test periods. Continual TTA over an entire procedure could instead use historical-state memory, parameter regularization, or multi-timescale update mechanisms to preserve useful adaptation while suppressing model drift and catastrophic forgetting.

Controlled degradation thus provides a reproducible foundation for analyzing TTA mechanisms, but the generalizability of the present findings still needs to be validated under more realistic shifts across devices, surgical procedures, and medical centers before the practical robustness of these methods in surgical robotic vision can be established.

### 6.6 From robust instrument segmentation to safe robotic perception

This study uses binary surgical instrument segmentation as the validation task and evaluates TTA primarily through DSC, NSD, and TC. In a surgical robotic system, however, segmentation often serves as a perceptual input to downstream tasks such as target tracking, pose estimation, interaction-region recognition, and assistive control. The value of TTA therefore cannot be assessed solely by pixel-level segmentation accuracy; it must also consider whether the perception state remains stable under continuing visual degradation and whether abnormal predictions can be detected in time.

From this perspective, an online-adaptation system must decide not only whether the model should be updated but also whether the adapted perception is sufficiently reliable for downstream robotic operation. When visual degradation is recoverable through online adjustment, the adapted model state can continue to be used. When abnormal predictions persist, the benefit of adaptation is uncertain, or the model state deviates substantially from a reliable range, further parameter updates should be restricted and a more conservative processing strategy should be adopted.

A promising direction is therefore an adapt-or-abstain mechanism for robotic deployment. Such a system could combine predictive uncertainty, adaptation-effectiveness estimates, historical stability, and anomaly detection to determine whether the current model output should continue to support downstream tasks. When perception is reliable, normal assistance or autonomous operation can continue; when reliability is insufficient, the system may freeze online parameters, restore a stable model state, reduce the level of automation, or request human confirmation. Compared with continually pursuing incremental gains in a single segmentation metric, an online perception system capable of detecting adaptation failure and executing safe fallback is more consistent with the reliability requirements of surgical robotics.

Future work should also extend TTA from an isolated visual-segmentation task to the full perception-decision chain. The practical effect of online adaptation could be assessed through instrument-tracking stability, pose-estimation error, interaction-region recognition, and downstream control performance. The value of TTA for intelligent medical robotics will be better established only when visual adaptation can reliably support downstream robotic tasks under complex intraoperative conditions while providing explicit anomaly detection and fallback mechanisms. Table 9 summarizes the principal empirical observations of this study and the corresponding directions for future research.

**TABLE 9:** Main empirical findings and corresponding future research directions.

Current issue	Experimental evidence	Future research direction
High predictive uncertainty does not necessarily imply an effective update	Under smoke, uncertainty and update rate remain high as severity increases, while DSC relative to source continues to decline	Adaptation-effectiveness-aware gating and update-utility estimation
The temporal term is coupled to the evolving update trajectory	Original LiteST-TTA exceeds selective entropy in 12/12 cells, but matched updates give 0.886967 vs. 0.886966 DSC with sequence-level p = 0.496	Update-budget-controlled temporal objectives and independent temporal-stability metrics
BN statistics account for most aggregate adaptation recovery	Statistics-only DSC = 0.886411; statistics + affine DSC = 0.887858; affine-only DSC = 0.847343	Normalization-centered low-cost adaptation and robustness under severe shift
Original main protocol contains checkpoint-selection overlap	A fixed 5/5 sequence-disjoint sensitivity analysis selects epoch 38 using 301 clean selection frames and evaluates 600 held-out frames; the dominant low-light pattern persists, with LiteST-TTA 0.8083 *versus* source 0.3251 under low-light L3, while source remains higher in 10/12 held-out cells	External cross-dataset validation and repeated sequence-level splits
The current protocol resets parameters at the video-sequence level	Online adaptation states are not retained across independent sequences	Continual TTA and anti-forgetting mechanisms over complete surgical procedures
Current evaluation focuses on instrument segmentation	Primary measures are DSC, NSD, TC, and computational metrics	Safety-oriented perception-decision evaluation for tracking, pose estimation, and control
Adaptation benefit depends on degradation type × severity	Sequence-level repeated-measures ANOVA: ΔDSC type × severity interaction corrected p < 0.001, partial η2 = 0.893	Factor-aware adaptation policies and validation under natural domain shifts

Overall, test-time adaptation for robotic surgical vision should consider the current state of the source model, the nature of the visual degradation, update effectiveness, the reliability of temporal supervision, and computational constraints together. The present experiments suggest that online adaptation is most useful when the source model has already undergone meaningful performance degradation, whereas increased uncertainty alone does not guarantee that parameter adjustment will be beneficial. Future research should improve the reliability of update decisions, reduce the cost of temporal modeling, and validate adaptation under real cross-domain and long-duration surgical conditions, with the eventual goal of online robotic perception systems that combine anomaly detection, controlled adaptation, and safe fallback.

## 7 Conclusion

This study investigates test-time adaptation for unlabeled continuous surgical video under controlled visual degradation and proposes LiteST-TTA for robot-assisted minimally invasive surgery. The method combines Top-k predictive-entropy gating, motion-compensated historical information, a hybrid temporal objective, and parameter-efficient BN-affine optimization. Using EndoVis 2017, we evaluate Source, BN Adapt, TENT, Selective Entropy, and LiteST-TTA across Blood, Blur, Low-light, and Smoke at three explicitly defined degradation-specific severity levels, and analyze segmentation accuracy, boundary quality, temporal behavior, update frequency, and computational efficiency.

TENT achieves the highest macro-average DSC of 0.8881, while LiteST-TTA reaches 0.8879. Sequence-level repeated-measures analysis verifies that adaptation behavior is not uniform across the controlled degradations: the type-by-severity interaction is significant for absolute DSC (corrected p = 0.031) and for adaptation gain relative to Source (corrected p < 0.001). Severe low illumination is the clearest beneficial case, with Source DSC falling to 0.3686 and LiteST-TTA recovering to 0.8724, whereas several Blood and Smoke conditions remain better served by the fixed Source model.

Mechanism controls substantially qualify the attribution of these results. Although the original LiteST-TTA configuration is higher than Selective Entropy in all 12 degradation cells, a matched-update experiment in which both methods update exactly the same frames produces virtually identical macro-average DSC values (0.886967 *versus* 0.886966; sequence-level p = 0.496), indicating that the original difference cannot be assigned solely to the temporal-consistency term. Under a unified 12-condition component-ablation protocol, the rollback-disabled hybrid yields the highest observed DSC among the LiteST-TTA variants, but its aggregate advantage over the matched hybrid-with-rollback setting is only +0.000581 DSC and condition-wise sequence-level evidence is limited after multiplicity correction. BN decomposition further shows that statistics-only adaptation reaches 0.886411 DSC, while the joint statistics-plus-affine configuration reaches 0.887858; thus, test-time BN-statistics adaptation accounts for most of the aggregate recovery. TC is consequently interpreted as an optimization-related diagnostic, and a flow-independent frame-to-frame DSC variation measure is used as additional temporal evidence.

In summary, the study supports a more qualified view of test-time adaptation for robotic surgical vision: adaptation benefit is strongly conditioned by degradation type, severity, and source-model robustness, while normalization adaptation, update dynamics, and temporal regularization are coupled rather than fully separable. The current optical-flow-based LiteST-TTA implementation also incurs substantial latency relative to simpler normalization adaptation. Although the original 901-frame protocol contains overlap between checkpoint selection and evaluation content, an additional sequence-disjoint sensitivity analysis separates these stages at the complete-video level and preserves the dominant pattern: severe low-light adaptation remains strongly beneficial, whereas Source remains higher in most other held-out conditions. This reduces, but does not eliminate, concerns about model-selection dependence because the held-out analysis uses a single 5/5 split. Future work should therefore prioritize independent cross-dataset and cross-device validation, update-budget-controlled temporal comparisons, lower-cost normalization and temporal mechanisms, and adaptation-effectiveness-aware fallback strategies for reliable robotic perception.

## Data Availability

Publicly available datasets were analyzed in this study. This data can be found here: EndoVis 2017 Robotic Instrument Segmentation Challenge dataset, Grand Challenge. Available at: https://endovissub2017-roboticinstrumentsegmentation.grand-challenge.org/Data/. No accession number is applicable.
