# Supplementary material for: Test-time adaptation for surgical instrument segmentation under visual degradations: selective updating and temporal constraints
Source: Front Robot AI. 2026 Oct 23;13:1991989. doi: 10.3389/frobt.2026.1991989 (PMC13741304; doi:10.3389/frobt.2026.1991989)
Supplement: Supplementary file 2 [file Supplementaryfile1.docx]

**Supplementary Material**

Test-Time Adaptation for Surgical Instrument Segmentation under Visual Degradations: Selective Updating and Temporal Constraints

# Supplementary Table S1

Full condition-wise paired DSC statistics for LiteST-TTA versus the four comparison methods.

*ΔDSC is defined as LiteST-TTA minus the comparison method. Positive values favor LiteST-TTA. Frame-level Holm p values correct the 12 degradation-cell comparisons separately for each comparator. Sequence-level Wilcoxon tests use the 10 video sequences as paired units; both the raw sequence-level p value and its Holm-adjusted value over the same 12 cells are shown in the final column. These sequence-level tests are treated as sensitivity analyses because only 10 sequences are available and neighboring frames within a sequence are correlated.*

## S1-1. LiteST-TTA vs Source

| **Condition** | **ΔDSC** | **95% CI** | **dz** | **Wilcoxon p** | **Holm p** | **Paired t p** | **Seq. p / Holm p** |
| --- | --- | --- | --- | --- | --- | --- | --- |
| Blood L1 | -0.013940 | [-0.017835, -0.010044] | -0.2340 | 1.51e-08 | 7.57e-08 | 4.30e-12 | 0.0273 / 0.2188 |
| Blood L2 | -0.012959 | [-0.016759, -0.009159] | -0.2230 | 0.0026 | 0.0104 | 3.85e-11 | 0.0488 / 0.2441 |
| Blood L3 | -0.010699 | [-0.014346, -0.007052] | -0.1918 | 0.2336 | 0.2336 | 1.17e-08 | 0.2324 / 0.7734 |
| Blur L1 | -0.012729 | [-0.016585, -0.008874] | -0.2159 | 0.0081 | 0.0162 | 1.51e-10 | 0.0273 / 0.2188 |
| Blur L2 | -0.010429 | [-0.014989, -0.005868] | -0.1495 | 0.0043 | 0.0129 | 8.12e-06 | 0.2324 / 0.7734 |
| Blur L3 | +0.007426 | [+0.002379, +0.012472] | +0.0962 | 2.81e-27 | 2.25e-26 | 0.0040 | 0.1934 / 0.7734 |
| Low-light L1 | -0.006080 | [-0.009233, -0.002926] | -0.1260 | 6.67e-14 | 4.00e-13 | 0.0002 | 0.4922 / 0.7734 |
| Low-light L2 | +0.036499 | [+0.029895, +0.043104] | +0.3613 | 2.08e-70 | 2.29e-69 | 7.61e-26 | 0.0371 / 0.2227 |
| Low-light L3 | +0.503788 | [+0.484756, +0.522820] | +1.7307 | 1.20e-146 | 1.44e-145 | 4.16e-273 | 0.0020 / 0.0234 |
| Smoke L1 | -0.016983 | [-0.020981, -0.012985] | -0.2777 | 5.12e-26 | 3.58e-25 | 2.83e-16 | 0.0039 / 0.0430 |
| Smoke L2 | -0.022596 | [-0.026878, -0.018315] | -0.3451 | 1.28e-44 | 1.15e-43 | 7.89e-24 | 0.0039 / 0.0430 |
| Smoke L3 | -0.026852 | [-0.031271, -0.022433] | -0.3973 | 1.25e-63 | 1.25e-62 | 1.52e-30 | 0.0039 / 0.0430 |

## S1-2. LiteST-TTA vs BN Adapt

| **Condition** | **ΔDSC** | **95% CI** | **dz** | **Wilcoxon p** | **Holm p** | **Paired t p** | **Seq. p / Holm p** |
| --- | --- | --- | --- | --- | --- | --- | --- |
| Blood L1 | +0.001137 | [+0.000924, +0.001350] | +0.3494 | 1.04e-56 | 6.23e-56 | 2.36e-24 | 0.0273 / 0.1641 |
| Blood L2 | +0.001221 | [+0.000980, +0.001462] | +0.3311 | 3.78e-64 | 3.02e-63 | 3.73e-22 | 0.0039 / 0.0469 |
| Blood L3 | +0.001239 | [+0.000941, +0.001536] | +0.2719 | 6.21e-37 | 1.24e-36 | 1.11e-15 | 0.0078 / 0.0547 |
| Blur L1 | +0.001115 | [+0.000892, +0.001338] | +0.3265 | 3.32e-49 | 1.33e-48 | 1.28e-21 | 0.0977 / 0.2227 |
| Blur L2 | +0.001192 | [+0.000817, +0.001567] | +0.2078 | 3.55e-27 | 3.55e-27 | 6.78e-10 | 0.2031 / 0.2227 |
| Blur L3 | +0.001396 | [+0.001141, +0.001651] | +0.3578 | 1.76e-46 | 5.29e-46 | 2.15e-25 | 0.0391 / 0.1953 |
| Low-light L1 | +0.000958 | [+0.000781, +0.001135] | +0.3536 | 5.50e-53 | 2.75e-52 | 7.05e-25 | 0.0547 / 0.2188 |
| Low-light L2 | +0.000954 | [+0.000812, +0.001097] | +0.4374 | 1.25e-66 | 1.12e-65 | 3.69e-36 | 0.0039 / 0.0469 |
| Low-light L3 | +0.001212 | [+0.001002, +0.001422] | +0.3777 | 9.74e-59 | 6.82e-58 | 6.03e-28 | 0.0742 / 0.2227 |
| Smoke L1 | +0.001687 | [+0.001406, +0.001967] | +0.3933 | 1.37e-88 | 1.37e-87 | 5.26e-30 | 0.0039 / 0.0469 |
| Smoke L2 | +0.002091 | [+0.001823, +0.002359] | +0.5101 | 3.32e-111 | 3.65e-110 | 3.37e-47 | 0.0039 / 0.0469 |
| Smoke L3 | +0.003153 | [+0.002765, +0.003542] | +0.5303 | 5.62e-125 | 6.75e-124 | 1.87e-50 | 0.0039 / 0.0469 |

## S1-3. LiteST-TTA vs TENT

| **Condition** | **ΔDSC** | **95% CI** | **dz** | **Wilcoxon p** | **Holm p** | **Paired t p** | **Seq. p / Holm p** |
| --- | --- | --- | --- | --- | --- | --- | --- |
| Blood L1 | -0.000289 | [-0.000365, -0.000213] | -0.2489 | 1.82e-33 | 1.64e-32 | 1.87e-13 | 0.1055 / 1.0000 |
| Blood L2 | -0.000209 | [-0.000284, -0.000134] | -0.1827 | 5.33e-20 | 2.13e-19 | 5.44e-08 | 0.1602 / 1.0000 |
| Blood L3 | -0.000257 | [-0.000326, -0.000187] | -0.2419 | 4.20e-20 | 2.10e-19 | 8.27e-13 | 0.5566 / 1.0000 |
| Blur L1 | -0.000297 | [-0.000391, -0.000203] | -0.2064 | 9.09e-17 | 1.24e-16 | 8.81e-10 | 0.2324 / 1.0000 |
| Blur L2 | -0.000222 | [-0.000286, -0.000159] | -0.2296 | 6.18e-17 | 1.24e-16 | 1.03e-11 | 0.7695 / 1.0000 |
| Blur L3 | -0.000322 | [-0.000412, -0.000233] | -0.2359 | 1.38e-38 | 1.38e-37 | 2.89e-12 | 0.0371 / 0.4082 |
| Low-light L1 | -0.000293 | [-0.000365, -0.000222] | -0.2695 | 1.54e-31 | 1.08e-30 | 1.92e-15 | 0.3750 / 1.0000 |
| Low-light L2 | -0.000301 | [-0.000366, -0.000236] | -0.3041 | 9.02e-40 | 9.93e-39 | 4.43e-19 | 0.1602 / 1.0000 |
| Low-light L3 | -0.000452 | [-0.000516, -0.000387] | -0.4556 | 1.26e-84 | 1.51e-83 | 8.10e-39 | 0.0273 / 0.3281 |
| Smoke L1 | -0.000254 | [-0.000305, -0.000203] | -0.3247 | 1.17e-31 | 9.34e-31 | 2.12e-21 | 0.1309 / 1.0000 |
| Smoke L2 | -0.000282 | [-0.000345, -0.000220] | -0.2958 | 3.27e-29 | 1.96e-28 | 3.57e-18 | 0.3594 / 1.0000 |
| Smoke L3 | -0.000191 | [-0.000248, -0.000134] | -0.2179 | 3.20e-18 | 9.60e-18 | 1.02e-10 | 0.2754 / 1.0000 |

## S1-4. LiteST-TTA vs Selective Entropy

| **Condition** | **ΔDSC** | **95% CI** | **dz** | **Wilcoxon p** | **Holm p** | **Paired t p** | **Seq. p / Holm p** |
| --- | --- | --- | --- | --- | --- | --- | --- |
| Blood L1 | +0.000814 | [+0.000653, +0.000975] | +0.3301 | 1.53e-49 | 9.17e-49 | 4.86e-22 | 0.0391 / 0.2734 |
| Blood L2 | +0.000829 | [+0.000643, +0.001014] | +0.2923 | 6.61e-53 | 5.29e-52 | 8.43e-18 | 0.0273 / 0.2461 |
| Blood L3 | +0.000999 | [+0.000746, +0.001253] | +0.2579 | 2.23e-36 | 4.45e-36 | 2.63e-14 | 0.0391 / 0.2734 |
| Blur L1 | +0.000912 | [+0.000720, +0.001104] | +0.3108 | 5.85e-46 | 2.34e-45 | 8.02e-20 | 0.0742 / 0.3711 |
| Blur L2 | +0.000973 | [+0.000702, +0.001244] | +0.2348 | 5.39e-34 | 5.39e-34 | 3.60e-12 | 0.1289 / 0.3867 |
| Blur L3 | +0.001055 | [+0.000845, +0.001265] | +0.3287 | 7.16e-43 | 2.15e-42 | 7.15e-22 | 0.1289 / 0.3867 |
| Low-light L1 | +0.000767 | [+0.000624, +0.000909] | +0.3518 | 7.93e-50 | 5.55e-49 | 1.17e-24 | 0.0742 / 0.3711 |
| Low-light L2 | +0.000821 | [+0.000699, +0.000944] | +0.4380 | 4.95e-67 | 4.45e-66 | 3.02e-36 | 0.0273 / 0.2461 |
| Low-light L3 | +0.001112 | [+0.000921, +0.001303] | +0.3808 | 2.61e-47 | 1.31e-46 | 2.40e-28 | 0.1641 / 0.3867 |
| Smoke L1 | +0.001337 | [+0.001103, +0.001571] | +0.3738 | 2.81e-85 | 2.81e-84 | 1.93e-27 | 0.0117 / 0.1172 |
| Smoke L2 | +0.001663 | [+0.001437, +0.001890] | +0.4795 | 9.76e-110 | 1.07e-108 | 1.97e-42 | 0.0039 / 0.0469 |
| Smoke L3 | +0.002719 | [+0.002347, +0.003090] | +0.4787 | 2.13e-120 | 2.56e-119 | 2.68e-42 | 0.0039 / 0.0469 |

# Supplementary Table S2

Condition-wise flow-independent frame-to-frame DSC variation. Values are mean ± SD of |DSCt−DSCt−1| computed only within video sequences; lower values indicate smaller frame-to-frame accuracy fluctuations.

*Note: Each condition contains 891 within-sequence frame transitions (901 frames across 10 sequences minus one first frame per sequence). No difference is computed across a sequence boundary.*

| **Method** | **Condition** | **\|DSCₜ−DSCₜ₋₁\| mean ± SD** |
| --- | --- | --- |
| Source | Blood L1 | 0.02376 ± 0.04458 |
| Source | Blood L2 | 0.03554 ± 0.05317 |
| Source | Blood L3 | 0.05791 ± 0.07552 |
| Source | Blur L1 | 0.02156 ± 0.04187 |
| Source | Blur L2 | 0.02732 ± 0.05681 |
| Source | Blur L3 | 0.04384 ± 0.08918 |
| Source | Low-light L1 | 0.02450 ± 0.04917 |
| Source | Low-light L2 | 0.06220 ± 0.11151 |
| Source | Low-light L3 | 0.22509 ± 0.20304 |
| Source | Smoke L1 | 0.02547 ± 0.04546 |
| Source | Smoke L2 | 0.03155 ± 0.05429 |
| Source | Smoke L3 | 0.03162 ± 0.06503 |
| BN Adapt | Blood L1 | 0.03317 ± 0.07011 |
| BN Adapt | Blood L2 | 0.04302 ± 0.07161 |
| BN Adapt | Blood L3 | 0.06071 ± 0.08231 |
| BN Adapt | Blur L1 | 0.03015 ± 0.06959 |
| BN Adapt | Blur L2 | 0.03320 ± 0.07281 |
| BN Adapt | Blur L3 | 0.03676 ± 0.07479 |
| BN Adapt | Low-light L1 | 0.02952 ± 0.06938 |
| BN Adapt | Low-light L2 | 0.03137 ± 0.07127 |
| BN Adapt | Low-light L3 | 0.04603 ± 0.07171 |
| BN Adapt | Smoke L1 | 0.03768 ± 0.07414 |
| BN Adapt | Smoke L2 | 0.04678 ± 0.08248 |
| BN Adapt | Smoke L3 | 0.04984 ± 0.07663 |
| TENT | Blood L1 | 0.03268 ± 0.06870 |
| TENT | Blood L2 | 0.04232 ± 0.07026 |
| TENT | Blood L3 | 0.06004 ± 0.08151 |
| TENT | Blur L1 | 0.02960 ± 0.06825 |
| TENT | Blur L2 | 0.03254 ± 0.07161 |
| TENT | Blur L3 | 0.03596 ± 0.07419 |
| TENT | Low-light L1 | 0.02909 ± 0.06832 |
| TENT | Low-light L2 | 0.03092 ± 0.07037 |
| TENT | Low-light L3 | 0.04549 ± 0.07118 |
| TENT | Smoke L1 | 0.03670 ± 0.07339 |
| TENT | Smoke L2 | 0.04544 ± 0.08183 |
| TENT | Smoke L3 | 0.04816 ± 0.07595 |
| Selective Entropy | Blood L1 | 0.03302 ± 0.06972 |
| Selective Entropy | Blood L2 | 0.04284 ± 0.07118 |
| Selective Entropy | Blood L3 | 0.06061 ± 0.08224 |
| Selective Entropy | Blur L1 | 0.03008 ± 0.06932 |
| Selective Entropy | Blur L2 | 0.03298 ± 0.07241 |
| Selective Entropy | Blur L3 | 0.03660 ± 0.07454 |
| Selective Entropy | Low-light L1 | 0.02946 ± 0.06923 |
| Selective Entropy | Low-light L2 | 0.03126 ± 0.07109 |
| Selective Entropy | Low-light L3 | 0.04599 ± 0.07163 |
| Selective Entropy | Smoke L1 | 0.03748 ± 0.07408 |
| Selective Entropy | Smoke L2 | 0.04658 ± 0.08232 |
| Selective Entropy | Smoke L3 | 0.04978 ± 0.07667 |
| LiteST-TTA | Blood L1 | 0.03277 ± 0.06896 |
| LiteST-TTA | Blood L2 | 0.04242 ± 0.07047 |
| LiteST-TTA | Blood L3 | 0.06020 ± 0.08171 |
| LiteST-TTA | Blur L1 | 0.02974 ± 0.06861 |
| LiteST-TTA | Blur L2 | 0.03265 ± 0.07176 |
| LiteST-TTA | Blur L3 | 0.03614 ± 0.07436 |
| LiteST-TTA | Low-light L1 | 0.02921 ± 0.06858 |
| LiteST-TTA | Low-light L2 | 0.03103 ± 0.07053 |
| LiteST-TTA | Low-light L3 | 0.04566 ± 0.07109 |
| LiteST-TTA | Smoke L1 | 0.03682 ± 0.07348 |
| LiteST-TTA | Smoke L2 | 0.04561 ± 0.08192 |
| LiteST-TTA | Smoke L3 | 0.04828 ± 0.07596 |

# Supplementary Table S3

Paired DSC comparison of final Hybrid − rollback (LiteST-TTA) versus Hybrid + rollback under the unified 12-condition protocol.

*Note: ΔDSC is LiteST-TTA minus Hybrid + rollback. Positive values favor rollback disabled. Holm-adjusted values are shown for the 12 condition-wise tests. The final aggregate row summarizes all 10,812 paired frame evaluations and the corresponding 10-sequence aggregate comparison.*

| **Condition** | **ΔDSC** | **95% CI** | **dz** | **Frame Wilcoxon / Holm p** | **Paired t p** | **Seq. Wilcoxon / Holm p** |
| --- | --- | --- | --- | --- | --- | --- |
| Blood L1 | +0.000592 | [+0.000475, +0.000709] | +0.3316 | 4.75e-41 / 2.85e-40 | 3.23e-22 | 0.0371 / 0.3711 |
| Blood L2 | +0.000612 | [+0.000480, +0.000743] | +0.3047 | 2.62e-50 / 1.83e-49 | 3.82e-19 | 0.0391 / 0.3711 |
| Blood L3 | +0.000521 | [+0.000369, +0.000672] | +0.2248 | 6.01e-24 / 1.20e-23 | 2.67e-11 | 0.3594 / 0.7188 |
| Blur L1 | +0.000508 | [+0.000400, +0.000615] | +0.3080 | 2.50e-36 / 1.00e-35 | 1.67e-19 | 0.1289 / 0.5156 |
| Blur L2 | +0.000441 | [+0.000268, +0.000615] | +0.1663 | 4.09e-11 / 4.09e-11 | 7.21e-07 | 0.4922 / 0.7188 |
| Blur L3 | +0.000560 | [+0.000452, +0.000669] | +0.3368 | 3.79e-31 / 1.14e-30 | 7.83e-23 | 0.1289 / 0.5156 |
| Low-light L1 | +0.000454 | [+0.000352, +0.000556] | +0.2903 | 4.62e-37 / 2.31e-36 | 1.40e-17 | 0.0547 / 0.3711 |
| Low-light L2 | +0.000477 | [+0.000392, +0.000562] | +0.3685 | 9.62e-52 / 7.70e-51 | 9.40e-27 | 0.0645 / 0.3711 |
| Low-light L3 | +0.000911 | [+0.000801, +0.001021] | +0.5423 | 1.65e-78 / 1.98e-77 | 2.03e-52 | 0.0098 / 0.1074 |
| Smoke L1 | +0.000612 | [+0.000520, +0.000703] | +0.4392 | 3.94e-60 / 3.55e-59 | 2.02e-36 | 0.0488 / 0.3711 |
| Smoke L2 | +0.000693 | [+0.000605, +0.000781] | +0.5133 | 1.75e-78 / 1.98e-77 | 1.01e-47 | 0.0039 / 0.0469 |
| Smoke L3 | +0.000598 | [+0.000510, +0.000686] | +0.4434 | 2.77e-66 / 2.77e-65 | 5.05e-37 | 0.0371 / 0.3711 |
| All 12 conditions | +0.000581 | [+0.000548, +0.000615] | +0.3278 | <1e-300 / <1e-300 | 6.47e-242 | 0.0273 / 0.0273 |
